# Health and Economic Impacts of Implementing Produce Prescription Programs for Diabetes in the United States: A Microsimulation Study

**DOI:** 10.1161/JAHA.122.029215

**Published:** 2023-07-07

**Authors:** Lu Wang, Brianna N. Lauren, Kurt Hager, Fang Fang Zhang, John B. Wong, David D. Kim, Dariush Mozaffarian

**Affiliations:** ^1^ The Gerald J. and Dorothy R. Friedman School of Nutrition Science and Policy Tufts University Boston MA USA; ^2^ Division of Clinical Decision Making Tufts Medical Center Boston MA USA; ^3^ Division of Hospital Medicine, Department of Medicine University of Chicago IL USA; ^4^ Division of Cardiology Tufts Medical Center Boston MA USA

**Keywords:** cost‐effectiveness, food is medicine, micro‐simulation, produce prescription, Cardiovascular Disease, Lifestyle, Primary Prevention, Secondary Prevention

## Abstract

**Background:**

Produce prescription programs, providing free or discounted produce and nutrition education to patients with diet‐related conditions within health care systems, have been shown to improve dietary quality and cardiometabolic risk factors. The potential impact of implementing produce prescription programs for patients with diabetes on long‐term health gains, costs, and cost‐effectiveness in the United States has not been established.

**Methods and Results:**

We used a validated state‐transition microsimulation model (Diabetes, Obesity, Cardiovascular Disease Microsimulation model), populated with national data of eligible individuals from the National Health and Nutrition Examination Survey 2013 to 2018, further incorporating estimated intervention effects and diet‐disease effects from meta‐analyses, and policy‐ and health‐related costs from published literature. The model estimated that over a lifetime (mean=25 years), implementing produce prescriptions in 6.5 million US adults with both diabetes and food insecurity (lifetime treatment) would prevent 292 000 (95% uncertainty interval, 143 000–440 000) cardiovascular disease events, generate 260 000 (110000–411 000) quality‐adjusted life‐years, cost $44.3 billion in implementation costs, and save $39.6 billion ($20.5–58.6 billion) in health care costs and $4.8 billion ($1.84–$7.70 billion) in productivity costs. The program was highly cost effective from a health care perspective (incremental cost‐effectiveness ratio: $18 100/quality‐adjusted life‐years) and cost saving from a societal perspective (net savings: $−0.05 billion). The intervention remained cost effective at shorter time horizons of 5 and 10 years. Results were similar in population subgroups by age, race or ethnicity, education, and baseline insurance status.

**Conclusions:**

Our model suggests that implementing produce prescriptions among US adults with diabetes and food insecurity would generate substantial health gains and be highly cost effective.

**Nonstandard Abbreviations and Acronyms**
SNAP: Supplemental Nutrition Assistance Program
WIC: Women, Infants, and Children program

**Clinical Perspective**

**What Is New?**

- Our validated microsimulation model estimates that produce prescription programs implemented nationally for US adults aged 40 to 79 years with diabetes and food insecurity could prevent 292 000 cardiovascular disease events, gain 260 000 quality‐adjusted life‐years, and save $39.6 billion in health care costs and $4.8 billion in productivity costs over a lifetime.
- Accounting for the food and administrative costs of the program, the intervention was highly cost effective from a health perspective, with an incremental cost‐effectiveness ratio of $18 100/quality‐adjusted life‐years, and cost saving from a societal perspective.
- The program remained cost effective when evaluated at shorter time horizons of 5 and 10 years.

**What Are the Clinical Implications?**

- Our new findings support the testing, scaling, and evaluation of produce prescription programs for patients with diabetes and food insecurity for both public and private payers, with a focus on ensuring access for those with the greatest need.
- In practice, a national produce prescription program could be enacted through the inclusion of produce prescriptions as a covered benefit, as is already occurring in limited capacities in select Medicare Advantage plans and in several state Medicaid programs, or perhaps through a new program administered by the US Department of Agriculture similar to the Supplemental Nutrition Assistance Program for Women, Infants and Children.

Poor diet is a leading risk factor for cardiometabolic diseases,^1^ contributing to nearly half of all US cardiometabolic deaths^2^ and high health care use and economic costs.^3^ These challenges disproportionately affect low‐income Americans, who often suffer from food and nutrition insecurity or lack of consistent access, availability, and affordability of nutritious foods that support their health and well‐being,^4^ resulting in significant health disparities.^2^, ^5^, ^6^ These challenges highlight the need for effective interventions to improve nutrition security and diet‐related health outcomes and disparities.

Until recently, health care providers have had few tools to improve diets in their patients. Produce prescription programs have emerged as a promising “Food is Medicine” health system strategy to improve nutrition, health outcomes, and health disparities in high‐risk patients with nutrition‐sensitive conditions, especially diabetes.^7^ In these programs, health care providers or payers first identify patients with 1 or more diet‐related health risks or conditions, often with additional criteria of food insecurity or other suboptimal access to nutritious foods, and then provide them with free or discounted healthy produce, most commonly fresh fruits and vegetables.^8^ This can be implemented using a paper voucher or electronic cards redeemable at local farmers' markets or retail grocery stores or with food packages picked up at a health care center or delivered to the home, with costs covered by health care payers. Typically, programs also include individual or group‐based nutrition education and recipe/cooking guidance.^8^, ^9^

A recent systematic review and meta‐analysis of 13 predominantly US‐based produce prescription interventions found these programs increased fruit and vegetable consumption by 0.8 servings per day, decreased body mass index (BMI) by 0.6 kg/m^2^, and among patients with diabetes, decreased hemoglobin A1c (HbA1c) by 0.8 points.^9^ Our recent pooled, patient‐level analysis of 9 other produce prescription programs across 22 sites in 12 states demonstrated similar health gains (Kurt Hager, PhD, unpublished data, 2023). These studies suggest that such programs represent an important opportunity to improve health outcomes and reduce health care usage and costs. In individual studies, the most consistent benefits have been observed among patients with type 2 diabetes, a condition creating high health needs and with rapidly increasing prevalence and health care costs. Therefore, identifying effective and cost‐effective new treatments for this population is a high priority.^10^, ^11^ However, the potential impacts of produce prescriptions on long‐term health gains, costs, and cost‐effectiveness among patients with diabetes have not been evaluated, either within or outside of the United States. Understanding these potential effects can further inform ongoing discussions around produce prescriptions, including optional coverage by Medicare Advantage plans started in 2020^12^ and a growing number of state Medicaid 1115 and 1915(b) waivers for food‐based demonstration projects.^13^, ^14^

To elucidate the potential impact of nationwide expansion of produce prescriptions for US patients with diabetes and food insecurity, we used a validated microsimulation model populated with nationally representative data to estimate the potential impacts on health, costs, and cost‐effectiveness. The model also allowed us to estimate the impacts of alternative inputs, parameters, and assumptions and report outcomes stratified by age, race or ethnicity, education, and baseline insurance payer (Medicare, Medicaid, dual eligible, private, no insurance).

## Methods

### Study Design

The data inputs and R codes that led to the findings of this study are available on GitHub at https://github.com/food‐price/Produce‐Rx. We used the Diabetes, Obesity, Cardiovascular Disease Microsimulation Model, a validated individual‐level, health state‐transition microsimulation model, to estimate the potential health and economic impacts and cost‐effectiveness of implementing produce prescriptions nationally for US adults with diabetes and food insecurity (David D Kim, PhD, unpublished data, 2023). The model predicts the annual probability of transitions among different health states for each individual based on individual‐level risk factors, temporal population trends for the risk factors, and potential impact of simulated interventions (Figure S1). By tracking transitions between health states, the model jointly tracks changes in BMI and incidence and prevalent diabetes, cardiovascular disease (CVD), all‐cause mortality, quality‐adjusted life years (QALYs), and health care costs at the individual level for the modeled population. More details for the model development, validation, and data sources have been documented in a separate paper and also briefly described in Data S1 in the Supplemental materials, with key model data inputs shown in Tables S1 and S2.

By comparing the identical population with and without produce prescription receipt, the model calculates the incremental changes in health and costs of implementing the policy versus the status quo over a lifetime horizon. Shorter‐term horizons at 5 years and 10 years, possibly more relevant to decision‐makers, were also assessed. Analyses followed the recommendations of the Second Panel on Cost‐effectiveness in Health and Medicine.^15^ Model outputs include first and recurrent CVD events, QALYs, and health care expenditures and policy costs. To explore potential impacts on health equity, stratified analyses were performed by baseline age (40–64, 65+ years), sex as a biological variable, race or ethnicity (non‐Hispanic White, non‐Hispanic Black, Hispanic [including Mexican American and other Hispanic], and others [including non‐Hispanic Asian, other races, and multiracial individuals]), education (less than high school, high school diploma/general equivalency diploma, some college or above), and income (family income to poverty ratio <1.3, 1.3–2.99, 3+). Stratified analyses were also performed by baseline insurance coverage status, including Medicare, Medicaid, dual eligible, private insurance, or no insurance coverage.

This study used publicly available, deidentified data sets. Thus this study was exempt from institutional review board review, and subject consent was not applicable.

### Study Subjects and Measurements

We populated the model with individuals aged 40 to 79 years who had both prevalent diabetes and food insecurity from 3 cycles of the National Health and Nutrition Examination Survey (NHANES; cycles 2013–2018) who represent 6.5 million eligible US adults after adjusting for NHANES sampling weights. Individual‐level characteristics including sociodemographic characteristics, baseline health status, food security, cardiometabolic risk factors, and medication use were collected using standardized questionnaires, examinations, and laboratory measurements. Diabetes was defined based on self‐reported diabetes or any one of the 5 clinical criteria (ie, fasting glucose ≥126 mg/dL, 2‐hour plasma glucose ≥200 mg/dL, HbA1c ≥6.5%, or using medication for diabetes). Food insecurity was determined using the US Food Security Survey Module collected in NHANES questionnaires.^16^ Missing data (generally below 10% for the relevant variables; Table S3) were handled using multiple imputations by predictive means matching, generating 10 sets of imputed values based on all the relevant variables (including sociodemographic characteristics, baseline anthropometrics, health status, laboratory measurements, and medications). Model results were average across the 10 imputed data sets to handle the imputation‐related uncertainty. In the base‐case analyses, we modeled the scenario that all eligible individuals received the intervention. In sensitivity analyses, we assessed the potential policy impact when 50% of eligible individuals received the intervention.

### Effects of the Intervention

To estimate the intervention effect size of produce prescription program on dietary habits and cardiometabolic risk factors, we performed a new meta‐analysis of 20 produce prescription interventions, including 11 studies in a recent systematic review,^9^ 3 more studies identified through PubMed searches (details shown in Table S4),^17^, ^18^, ^19^ and 6 more studies from a new analysis of completed produce prescription programs by our research group (Kurt Hager, PhD, unpublished data, 2023). We focused on programs that evaluated the impact of produce prescriptions over at least 3 months on changes in fruit and vegetable intake, BMI, or HbA1c. Included studies were either quasiexperimental pre/post studies with or without a control group (n=17) or randomized controlled trials (n=3). These produce prescription programs were integrated into health care systems and enrolled adults with chronic, diet‐related health conditions or food insecurity identified or referred by a health care provider. Among the eligible studies, most (17 of 20) enrolled adults with poor cardiometabolic health including diabetes or prediabetes, hypertension, CVD, obesity, or overweight. All programs enrolled participants who were food insecure or at high risk for food insecurity, with a weighted average of 74% being food insecure. In addition, all 10 programs that assessed the effect on HbA1c were implemented among patients with diabetes, with 79% being food insecure.

Based on random effects meta‐analysis, the produce prescription programs increased average fruit and vegetable consumption by 0.80 servings/day (95% CI, 0.45–1.15), reduced BMI by 0.36 kg/m^2^ (95% CI, 0.16–0.55) per day, and reduced HbA1c by 0.63% (95% CI, 0.28–0.98) (Figures S2 through S4). Due to insufficient evidence for any differential effects, we did not make assumptions about differences in intervention efficacy by population subgroups. Of note, a recent study evaluating produce prescriptions identified no significant evidence of differential effects by age, sex, race or ethnicity, or enrollment status of the Supplemental Nutrition Assistance Program (SNAP).^20^ We assumed continuing enrollment in the intervention each year, with stable intervention effects over time, and conservatively assumed that all health benefits would end (ie, no persistent benefits) if an individual were to stop receiving produce prescriptions.

### Intervention Costs

Intervention costs included the food costs for fruits and vegetables and the administrative costs of program implementation. The food costs ($ per person per month) were calculated based on the weighted mean food box value or voucher amounts from 20 studies included in the intervention effect size meta‐analysis, adjusted for the proportion of fruits and vegetables in the food box (when other items were included) and the actual redemption rates of the vouchers (Table S4). The value of the reported incentive offered to participants in each study was converted into a standardized US dollar‐equivalent amount adjusted for inflation to 2021. The interventions offered a weighted mean of $42/month of fruits and vegetables to the targeted population; and after subtracting unused vouchers, patients redeemed a weighted mean of $32/month.

To estimate the administrative costs of implementing produce prescriptions, we reviewed the cost data of the Special Supplemental Nutrition Program for Women, Infants, and Children (WIC)^21^ and the SNAP.^22^ WIC and SNAP share similar administrative components with produce prescription programs, including personnel and training, eligibility certification, quality control, use of the electronic benefits transfer system or food delivery, benefit and retailer redemption and monitoring, nutrition education, and program evaluation. We assumed that the administrative costs of a national produce prescription program would be 15% of total program costs, or about 2‐3‐fold higher than the administrative costs of SNAP (5%–8%) and one‐third lower than the administrative costs of WIC (21.3%) given that produce prescriptions provide only produce rather than multiple categories of foods, do not require income certification, and do not involve establishment of separate clinical institutions (WIC clinics) (detailed estimation process described in Data S1). In sensitivity analysis, we evaluated alternative administrative costs of 8% (upper end of SNAP costs) and 21.3% (WIC costs). We also assumed the costs to be higher in the first year of implementation due to program launching, equal to 33% of the total program costs (50% of food costs).

### Effects on Cardiometabolic Outcomes

Our detailed methods for reviewing and synthesizing evidence to estimate effect sizes for associations between dietary factors and cardiometabolic end points and for confirming the validity of these findings have been reported.^23^, ^24^ Effects of changes in fruit and vegetable intake on CVD, obesity, and diabetes were estimated from published meta‐analyses of prospective cohorts or randomized clinical trials evaluating direct associations of fruit and vegetable consumption with coronary heart disease (CHD), stroke, weight gain, and diabetes by age.^2^, ^23^, ^24^ Because these observed effects assess how dietary differences relate to clinical risk in populations, they inherently incorporate the overall impact including the average dietary complements and substitutes in the population. All identified observational studies in the meta‐analyses used included multivariable adjustment for other risk factors to reduce bias from confounding. Although some studies included serial dietary measures, measurement error due to changes in diet over time was generally not addressed, which could cause underestimation of the true effect sizes. We identified and incorporated evidence for association between changes in fruit and vegetable consumption and CHD and stroke (Table S5).

### Health Care Costs

The simulation model incorporated a de novo prediction algorithm to estimate annual health care for each individual each year based on their unique characteristics, including age, sex, race or ethnicity, BMI, diabetes, hypertension, and history of CVD. The algorithm was developed using data from the 2014–2016 Medical Expenditure Panel Survey on 73 174 individuals, representing a weighted noninstitutionalized population of 244.4 million adults age 18+ years.^25^ Survey design and weights were applied to generate nationally representative estimates. Survey design and weights were applied to derive nationally representative estimates.^26^ The predicted health care costs represent the average sum of costs for office‐based visits, hospital outpatient visits, emergency room visits, inpatient hospital stays, prescription drugs, dental visits, and home care. The model further included event‐ and procedure‐specific costs from weighted‐average total payments of full discharges across relevant diagnosis‐related groups from the Medicare Inpatient Prospective Payment data.^27^ In addition to prevalence of diabetes, the model accounted for diabetes control as measured by HbA1c, an established determinant of health care costs among patients with diabetes independent of other comorbidities such as CVD and hypertension^28^, ^29^; and prevented double‐counting of benefits from fewer CVD outcomes by applying only the HbA1c‐related costs of diabetes excluding CVD‐dependent costs (details in Data S1). Productivity costs including costs of lost productivity from morbidity and premature mortality associated with prevalent CHD and stroke were estimated by dividing national estimates of total annual productivity costs of CHD and stroke in 2021 by the number of projected US CHD and stroke cases in 2021.^30^

### Health‐Related Quality of Life

To calculate QALYs, the model incorporated an established prediction model for the US population to estimate individual‐level health‐related quality of life measured by European Quality of Life‐5D questionnaire on a scale of 0.00 (death) to 1.00 (perfect health) based on demographic, socioeconomic, and chronic disease factors in each year.^31^ The model further incorporated event‐specific short‐term (1‐year) decrements in health‐related quality of life for individuals experiencing an acute CHD (−0.055) or stroke (−0.3) in a given year.^32^

### Statistical Analysis

Following recommendations from the Second Panel on Cost‐effectiveness in Health and Medicine,^33^ we conducted analyses from a health care perspective, incorporating intervention costs and health care cost‐savings, and from a societal perspective with additional consideration of savings in productivity costs due to reduced disease burden. All costs were inflated to 2021 US dollars, and costs and QALYs discounted annually by 3%. Net changes in costs from a health care perspective were calculated as intervention costs minus health care cost‐savings, and from a societal perspective, further subjected by savings in productivity costs. Incremental cost‐effectiveness ratios (ICERs) were calculated as the net change in costs divided by the net change in QALYs. Willingness‐to‐pay thresholds were evaluated at $150 000 and $50 000 per QALY, as recommended by the American College of Cardiology and American Heart Association.^34^

To jointly account for uncertainty in key model inputs, probabilistic sensitivity analyses incorporated uncertainty distributions for all input parameters including intervention effects, diet‐disease associations, intervention costs, health care costs, and health‐related quality of life, using 1000 Monte Carlo simulations. NHANES sampling weights were incorporated to provide estimates representative of the noninstitutionalized US population in each simulation. The 95% uncertainty intervals for model outcomes were estimated by jointly combining the sampling uncertainty of NHANES and the uncertainties of multiple input parameters using the Rubin's rule.^35^ All analyses were conducted in R, version 4.0.2.

We performed 1‐way sensitivity analyses to assess the impact of specific inputs and assumptions. We evaluated varying policy efficacy by varying the intervention effect sizes at the 2.5th, 10th, 25th, 50th, 75th, 90th, and 97.5th percentiles of changes in fruit and vegetable intake, BMI, and HbA1c. We evaluated alternative assumptions on administrative costs at 8% (upper end of SNAP costs) and 21.3% (WIC costs) of the total program costs; and estimated the potential policy impact of assuming 50% of eligible patients each year received produce prescription. Finally, a threshold analysis estimated the maximum monthly intervention costs for the program to remain cost effective (<$150 000/QALY).

## Results

### Baseline Characteristics and Dietary Intakes

Based on national data, 6.5 million US adults met eligibility criteria: age 40–79 years at baseline and having both diabetes and food insecurity (Table 1). Mean (SD) baseline age was 58.2 (10.2) years, fewer than half (43.1%) were non‐Hispanic White adults, nearly two‐thirds (63.0%) had a high school education or less, and more than half (56.6%) had a family income to poverty ratio lower than 1.3. Most were covered by Medicare (39.4%) at the baseline, which include those on Medicare only (26.9%) and dual eligible (12.5%), followed by private payers (29.4%) and Medicaid (26.9%). About 16.5% had no insurance coverage at the baseline. Nearly 1 in 3 (30.0%) had baseline prevalent CVD, mean (SD) BMI was 33.6 (7.95) kg/m^2^, and mean (SD) HbA1c was 7.3 (1.95). Baseline mean (SD) daily consumption of fruits and vegetables was 0.86 (1.08) servings of fruit and 1.30 (1.04) servings of vegetables.

**Table 1:** Baseline Characteristics of US Adults Aged 40 to 79 Years in the Model With Both Diabetes and Food Insecurity*

	Characteristics
No. of US adults represented†	6.5 million
Age, y, mean (SD)	58.2 (10.2)
Sex, percentage	
Female	55.6%
Race or ethnicity, percentage‡	
Non‐Hispanic White	43.1%
Non‐Hispanic Black	17.2%
Hispanic	29.0%
Other	10.7%
Educational level, percentage	
Less than high school graduate	37.1%
High school graduate§	26.5%
Some college or greater	36.4%
Family income to poverty ratio||, percentage	
<1.30	56.6%
1.30–1.84	17.1%
1.85–2.99	16.4%
>3.00	9.9%
Insurance payers	
Private insurance	29.4%
Medicare	26.9%
Medicaid	26.9%
Dual eligible	12.5%
No insurance coverage	16.5%
Baseline high blood pressure	74.8%
Baseline cardiovascular disease history, percentage	30.0%
Angina	15.8%
Coronary heart disease	11.9%
Myocardial infarction	12.8%
Stroke	9.1%
Baseline diabetes	100.0%
Baseline food insecurity	100.0%
Baseline fruit consumption, mean (SD)	0.86 (1.08)
Baseline vegetable consumption, mean (SD)	1.30 (1.04)
Baseline body mass index, kg/m^2^, mean (SD)	33.6 (7.95)
Baseline HbA1c, %, mean (SD)	7.3 (1.95)

HbA1c indicates hemoglobin A1c.

* The modeled sample was drawn from participants in combined cycles of the 2013 to 2018 National Health and Nutrition Examination Survey (NHANES), with a survey sample size of 843 adults with two 24‐hour dietary recalls meeting the criteria of diabetes (self‐reported diabetes, fasting glucose ≥126 mg/dL, 2‐hour plasma glucose ≥200 mg/dL, HbA1c≥6.5%, or using medication for diabetes) as well as low or very low food security. All analyses incorporated NHANES sampling and survey weights to be representative of national population of noninstitutionalized US adults.

^†^ Based on the survey weight‐adjusted population size of the modeled NHANES sample.

^‡^ Participants' reported information on race or ethnicity according to fixed categories. The “Other” group included non‐Hispanic Asian, and other races‐including multiracial individuals.

^§^ Including a general equivalency diploma.

^||^ The ratio of family income to the federal poverty threshold, adjusting for household size. For reference, the 2019 federal poverty threshold for a family of 4 was $25 750/year.

### Impact on Health Outcomes

Our simulation model projected that implementing produce prescriptions nationally that provided an average monthly voucher or food boxes of $42 per patient ($504/year) in this population, would prevent 292 000 CVD events (95% uncertainty interval, 143 000–440 000) and generate 260 000 additional QALYs (110 000–411 000) (Table 2) over a lifetime. At 5 years, the intervention would prevent 66 900 CVD events (31 900–102 000); and at 10 years, 126 000 CVD events (62 300–190 000).

**Table 2:** Estimated Health Gains, Costs, and Cost‐Effectiveness of a National Produce Prescription Program at 5 years, 10 years, and Over a Lifetime*

Outcome	Over 5 y		Over 10 y		Over lifetime (mean=25 y)	
	Estimated change with policy	95% UI	Estimated change with policy	95% UI	Estimated change with policy	95% UI
Health outcomes						
Total CVD events averted, thousands	66 900	(31 900 to 102 000)	126 000	(62 300 to 190 000)	292 000	(143 000 to 440 000)
First CVD cases averted, thousands	32 100	(14 800 to 49 400)	53 100	(25 200 to 80 900)	77 500	(35 500 to 120 000)
Recurrent CVD events averted, thousands	34 700	(15 800 to 53 700)	73 200	(35 400 to 111 000)	214 000	(105 000 to 323 000)
QALYs gained, thousands	19 800	(5810 to 33 900)	49 200	(16 000 to 82 500)	260 000	(110 000 to 411 000)
Health care cost savings, $ billions†	11.6	(6.2 to 17.0)	20.7	(11.1 to 30.3)	39.6	(20.5 to 58.6)
Productivity cost savings, $ billions	0.53	(0.215 to 0.814)	1.44	(0.59 to 2.29)	4.77	(1.84 to 7.70)
Intervention costs, $ billions						
Food costs	11.1	(7.98 to 14.1)	19.7	(14.3 to 25.0)	37.3	(27.0 to 47.5)
Administrative costs	2.37	(1.71 to 3.03)	3.89	(2.83 to 4.95)	6.99	(5.07 to 8.91)
Net change in costs, $ billions‡						
Health care perspective	1.84	(−4.84 to 8.52)	2.85	(−8.73 to 14.4)	4.72	(−17.5 to 26.9)
Societal perspective	1.31	(−5.41 to 8.03)	1.41	(−10.3 to 13.1)	−0.049	(−22.8 to 22.7)
Cost‐effectiveness						
ICER, health care perspective (dollar/QALY)§	92 700		58 000		18 100	
ICER, societal perspective (dollar/QALY)§	66 100		28 700		Cost‐saving	

CVD indicates cardiovascular disease; ICER, incremental cost‐effectiveness ratio; QALY, quality‐adjusted life‐years; and UI, uncertainty interval.

* Using a microsimulation model (Diabetes, Obesity, Cardiovascular Disease Microsimulation model), outcomes were evaluated in a nationally representative sample of US adults aged 40 to 79 years with diabetes and food insecurity at baseline, corresponding to 6.5 million adults, followed until death or 60 years of follow‐up, whichever occurred first. The intervention was a produce prescription program integrated into the health care system, with eligible patients identified and referred by a health care provider. All costs were inflated to constant 2021 US dollars using the Bureau of Labor Statistics' Consumer Price Index. All costs and QALYs were discounted by 3% annually. Estimations represent the mean value from 1000 Monte‐Carlo simulations.

^†^ Health care cost‐savings were calculated as averted direct costs from chronic/acute disease states, surgical procedures, screening, and drug use.

^‡^ Net changes in costs from a health care perspective=total intervention costs−health care cost‐savings by produce prescription. Net changes in costs from a societal perspective=total intervention costs−health care cost‐savings−savings from averted productivity loss. Negative values in the net costs indicate cost savings.

^§^ ICERs were calculated as the estimated mean net change in costs from a health care perspective and societal respectively divided by the mean net change in QALYs by produce prescriptions. ICERs below $100 000/QALY were considered cost effective.

### Costs and Cost‐Effectiveness

**Figure 1:**
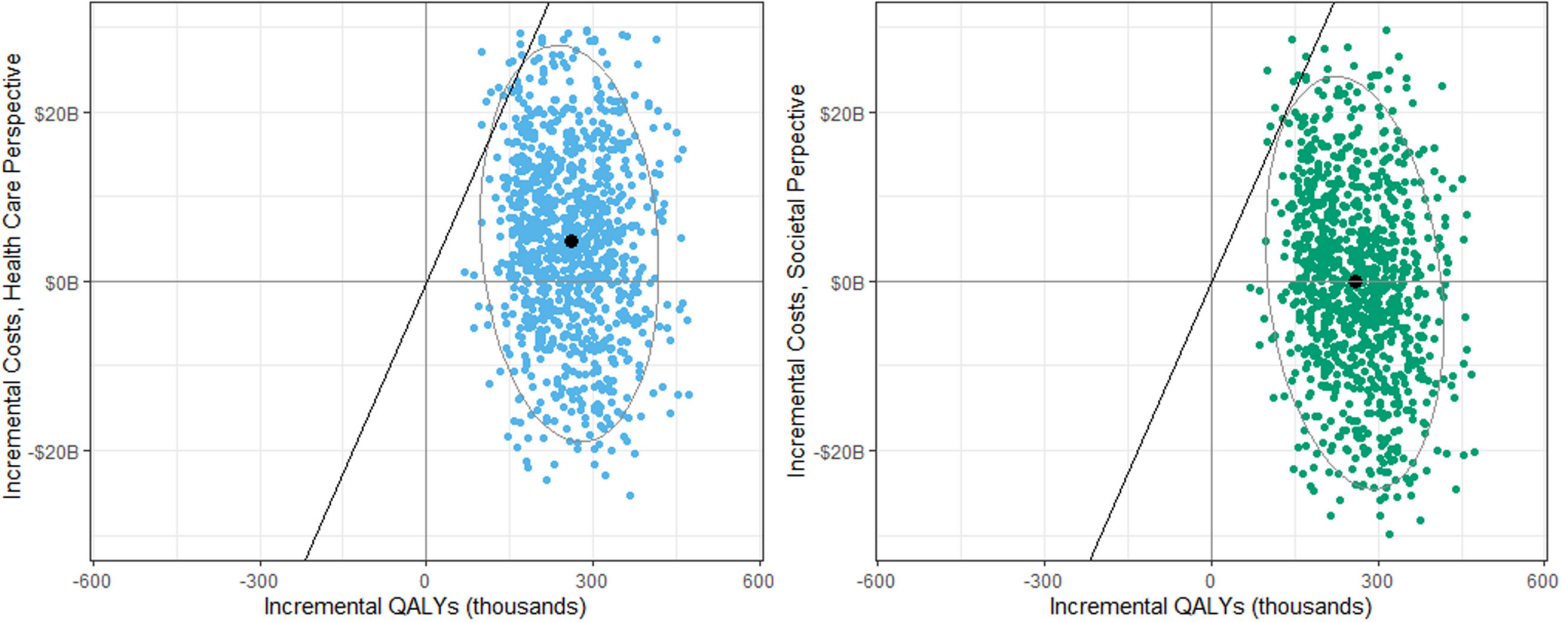
Probabilistic sensitivity analyses for the cost‐effectiveness of the national produce prescription programs over a lifetime. Note: Each point represents results from 1 of 1000 Monte Carlo simulations, and the ellipse depicts the 95% UIs across all simulations. The black point indicates the mean value for the 1000 simulations. The solid black line represents a value of $150 000/QALY. Over a lifetime, the probability of the produce prescription program being cost‐effective at a threshold of <150 000$/QALY was 98.3% from a health care perspective, and 98.9% from a societal perspective. B indicates billion; QALY, quality‐adjusted life year; and UI, uncertainty index.

Over a lifetime, produce prescriptions were estimated to cost $37.3 billion ($27.0–$47.5 billion) in food costs and $6.99 billion ($5.07–$8.19 billion) in administrative costs (Table 2). The intervention would save an estimated $39.6 billion ($20.5–$58.6 billion) in formal health care expenditures and $4.77 billion ($1.84 billion–$7.70 billion) in productivity costs. The program was highly cost effective from a health care perspective with an ICER of $16 900/QALY and cost saving from societal perspectives, with net savings of $0.47 billion ($23.7 saving–$23.5 billion cost). Over a lifetime, the probability of net cost‐effectiveness was 98.4% and 98.9% from a health care and societal perspective respectively, when evaluated at a $150 000 willingness‐to‐pay threshold, and 74.4% and 84.6%, respectively, when evaluated at the $50 000 willingness‐to‐pay threshold (Figure 1).

The intervention was also cost effective at shorter time horizons, with ICERs of $92 700/QALY at 5 years and $58 000/QALY at 10 years from the health care perspective and corresponding ICERs of $66 100/QALY and $28 700/QALY from the societal perspective.

### Subgroup Analyses

**Figure 2:**
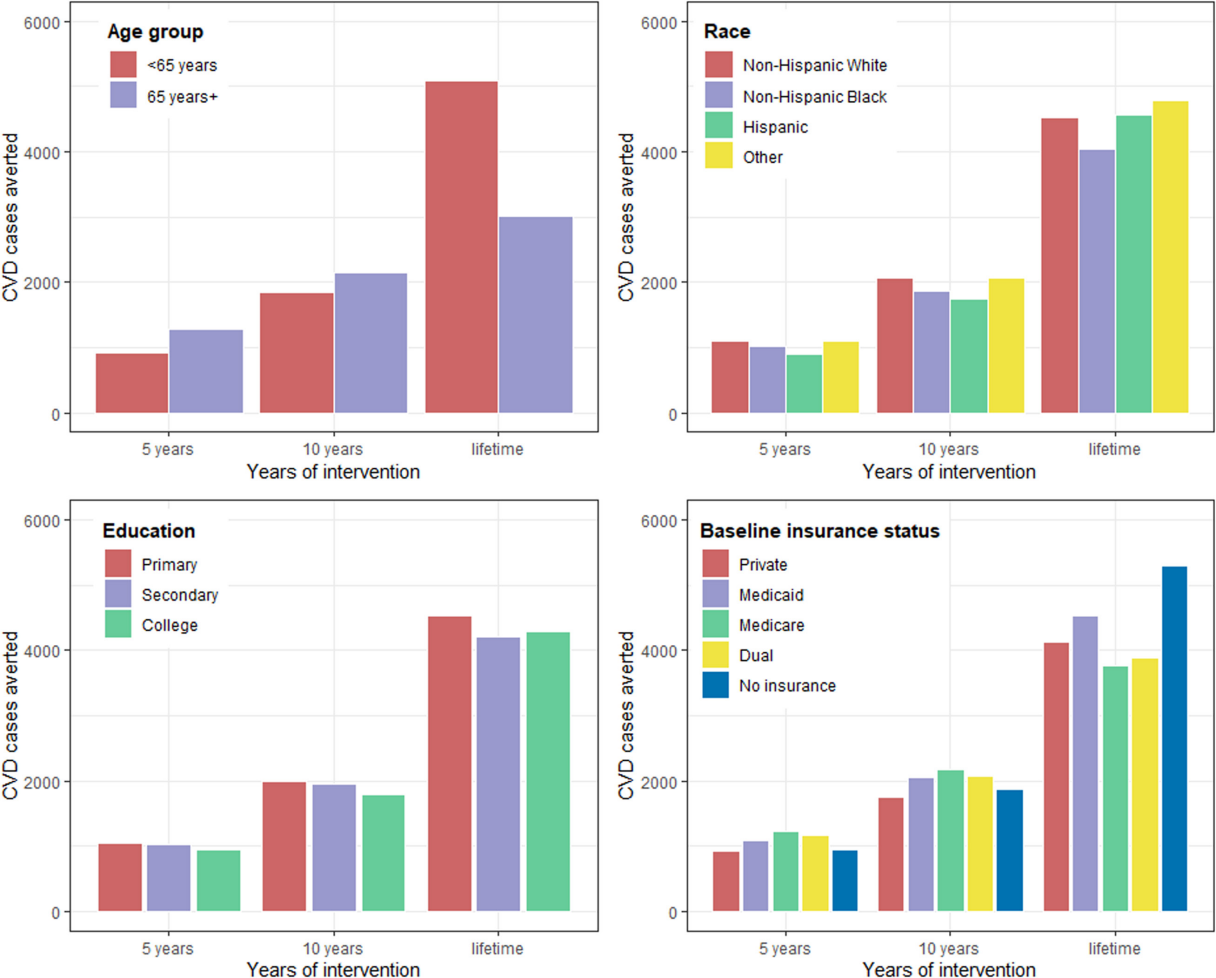
The number of CVD cases averted per 100 000 patients by produce prescriptions nationally for adults with diabetes and food security, by population subgroups at 5 years, 10 years, and lifetime. Bars represent the mean number of CVD cases (or first‐time CVD events) averted by produce prescriptions per 100 000 patients estimated from 1000 Monte‐Carlo simulations using the Diabetes, Obesity, Cardiovascular Disease Microsimulation model, by comparing the same population (patients with diabetes and food insecurity) with and without implementation of produce prescriptions. The “Other” race or ethnicity group includes non‐Hispanic Asian and all other races including multiracial individuals. CVD indicates cardiovascular disease.

Over a lifetime, the national produce prescription program averted more CVD cases and saved more QALYs and health care costs per 100 000 patients among those who were younger (<65 years) at baseline than those who were older (65+ years); and among non‐Hispanic Black and Hispanic patients than non‐Hispanic White patients and other races or ethnicities (Figure 2, and Figures S5 and S6). By payer, the most lifetime CVD cases per 100 000 were averted in patients with no insurance coverage at baseline, followed by those covered by Medicaid or private insurance at baseline. With shorter time horizons of 5 years and 10 years, more CVD cases were averted per 100 000 adults among those who were older (aged 65+ years) than younger, and among non‐Hispanic Black and Hispanic patients than patients with non‐Hispanic White and other racial or ethnic backgrounds. The program achieved similar health benefits per 100 000 patients among those with different education levels, at all time horizons.

**Figure 3:**
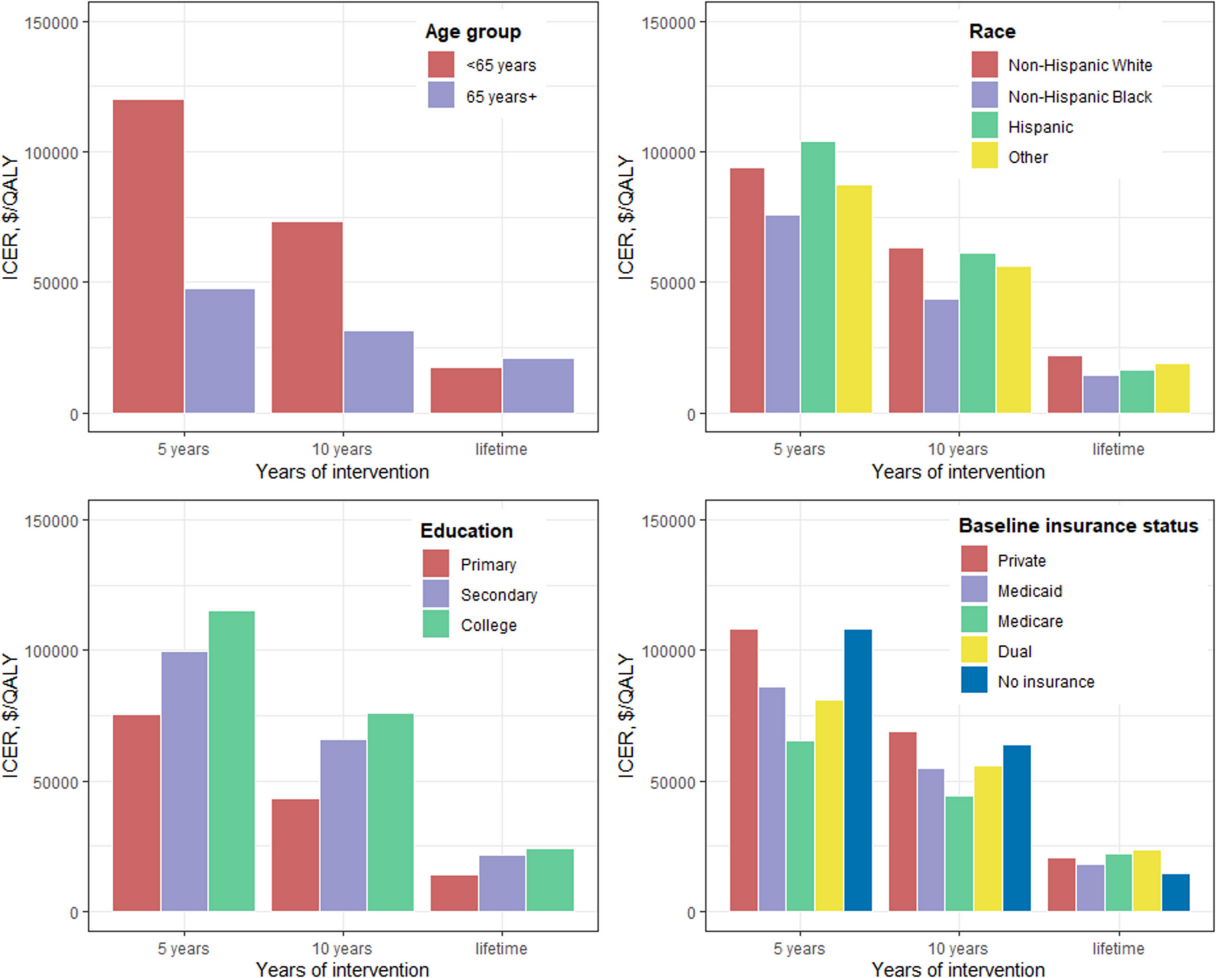
Estimated cost‐effectiveness of produce prescriptions nationally for adults with diabetes and food security, by population subgroups at 5 years, 10 years, and lifetime. Bars represent the ICER for implementing produce prescriptions among US adults with diabetes and food insecurity, from a health care perspective, calculated as the estimated mean net change in costs (intervention costs minus health‐related cost savings) divided by the mean net change in QALYs. The Diabetes, Obesity, Cardiovascular Disease Microsimulation model was used to project the health and economic outcomes for the same population with and without implementing produce prescriptions, based on 1000 Monte‐Carlo simulations. The “Other” race or ethnicity group includes non‐Hispanic Asian and all other races including multiracial individuals. ICER indicates incremental cost‐effective ratio; and QALY, quality‐adjusted life year.

The program was cost effective over a lifetime of intervention among all population subgroups by age, race or ethnicity, education, and baseline insurance payer (Figure 3), with similar ICERs achieved. With shorter time horizons of 5 years and 10 years, a lower ICER (ie, greater cost‐effectiveness) was observed for older (65+ years) versus younger patients, and among those covered by Medicare only or Medicaid only versus those covered by private payers or uninsured at the baseline.

### Sensitivity Analysis

In 1‐way sensitivity analyses, the national produce prescriptions remained cost‐effective (<$150 000/QALY) over a lifetime when varying the intervention effects size at the 2.5th, 10th, 25th, 50th, 75th, 90th, and 97.5th percentiles of its distribution, from both health care and societal perspectives, with ICERs ranging from cost‐saving (at 75th percentile or higher) to $102 000/QALY (at the 2.5th percentile) (Figure S7). The estimated lifetime net costs from a health care perspective varied from $15.3 billion in savings at the 97.5th percentile of the effect size distribution to $24.1 billion in additional cost at the 2.5th percentile.

When varying assumptions on administrative costs, the program was cost effective or cost saving over a lifetime when administrative costs were lower at 8% of total program costs ($9300/QALY in health care perspective, cost‐saving in societal perspective) or higher at 23% of total program costs ($36 800/QALY and $18 500/QALY, respectively) (Figure S8). If only 50% of eligible patients participated in the program each year, the program remained highly cost effective, with net costs of $5.86 billion and $3.47 billion over a lifetime intervention from a health care and societal perspective with corresponding ICERs of $450 000 /QALY and $26 700/QALY, respectively. Threshold analyses suggested that the program would be cost effective at lifetime until the per patient intervention cost exceeded $67.2 per month in total redemptions and administrative costs.

## Discussion

By incorporating nationally representative data and evidence from meta‐analysis, our validated microsimulation model estimates that produce prescription programs implemented nationally for US adults aged 40 to 79 years with diabetes and food insecurity could prevent 292 000 CVD events, gain 260 000 QALYs, and save $39.6 billion in health care costs and $4.8 billion in productivity costs over a lifetime. Accounting for the food and administrative costs of the program, the intervention was highly cost effective from a health perspective, with an ICER of $18 100/QALY, and cost saving from a societal perspective. The program remained cost effective when evaluated at shorter time horizons of 5 and 10 years. Our results further suggested that this program would generate health benefits and be similarly cost‐effective for all subgroups of eligible patients evaluated, including by age, race or ethnicity, education, and baseline insurance coverage status.

Produce prescription programs are designed to treat patients with diet‐sensitive diseases and often (although not always) with coexisting food insecurity. Compared with the general population, the eligible Americans we identified were more likely to be in racial or ethnic minority groups and have lower income and education levels. Therefore, produce prescriptions could help address health inequities associated with food and nutrition insecurity and diet‐related diseases. Our results suggest larger per person health benefits among non‐Hispanic Black and Hispanic patients than non‐Hispanic White patients, owning to worse baseline cardiometabolic risk profiles and higher mortality risk in these groups. However, efforts are needed to realize this potential by ensuring equitable access to produce prescriptions, given the disparate challenges for different population subgroups around health care access and quality of care.^36^ Related to this, 16% of eligible patients in our analysis did not have insurance coverage at baseline. Although many of these patients might obtain employer‐based coverage over their lifetimes, and Medicare after age 65, the larger lifetime health benefits of produce prescriptions in this group highlight the importance of continuing efforts to expand insurance coverage to more Americans.

In a prior national simulation study, we estimated the impact of subsidizing fruit and vegetable purchases for all patients covered by Medicare/Medicaid, using a simple electronic benefits transfer card that provided a 30% subsidy at the point of sale.^37^ That general electronic benefits transfer‐based subsidy program, estimated to cost about $110 per person per year, was far less intensive and expensive than produce prescriptions (which involve screening, provision of vouchers and in some cases delivery of food, and nutrition education) and was estimated to be cost effective ($14 600/QALY)^37^, ^38^ from a health care perspective. However, the estimated changes in fruit and vegetable consumption in that earlier study were derived from a meta‐analysis of food price interventions, rather than directly identified effects of produce prescription programs (which we have now used and identified a smaller relative change in fruit and vegetable intake per dollar) and also assumed lower administrative costs. The present analysis incorporates new evidence from produce prescription programs on target patient populations, changes in fruit and vegetable intake, BMI, and HbA1c and food redemption and administrative costs; uses a validated microsimulation model that jointly assesses and integrates changes in lifestyle, risk factors, obesity, diabetes, and CVD; and provides a more nuanced and accurate assessment of implementing produce prescription programs among patients with diabetes and food insecurity. The identified cost‐effectiveness ($18 100/QALY from a health care perspective) is similar in magnitude to other covered primary prevention strategies based on drug treatment^39^ such as drug treatment for hypertension ($20 000/QALY)^40^ or use of statins for primary prevention ($37 000/QALY).^41^

Our findings are timely and relevant given the rapid growth of interest in produce prescription programs. The US Department of Agriculture recently announced a $59.4 million expansion of produce prescription programs through the Gus Schumacher Nutrition Incentive Program's Produce Prescription and Nutrition Incentive programs.^42^ Private health care payers like Geisinger Health are expanding their produce prescription programs, with a focus on patients with diabetes and food insecurity. The National Produce Prescription Collaborative brings together providers of produce prescriptions across the country to share best practices and identify opportunities for incorporating this intervention within health care payment models.^43^ Produce prescriptions are being implemented for patients in North Carolina and Massachusetts under their 1115 Medicaid waivers; and in California under a new 1115/1915(b) Medicaid waiver. On September 28, 2022, the White House announced a new National Strategy on Hunger, Nutrition and Health, including a major focus on testing produce prescription programs in Medicaid, Medicare, Veterans Affairs, and the Indian Health Service.^44^ As part of the National Strategy, Kaiser Permanente also committed $50 million for new food is medicine programs, the American Heart Association and Rockefeller Foundation committed $250 million for more research in food is medicine, the American Academy of Pediatrics committed to training all its 67 000 member pediatricians on screening for nutrition insecurity and referring patients to nutrition resources, and the American College of Lifestyle Medicine committed to training 100 000 health care providers on food is medicine interventions in regions with high rates of diet‐related disease.^45^ Despite this growing interest, today produce prescriptions reach only a minority of Americans, with limited access by most health care providers or eligible patients. Our new findings support the testing, scaling, and evaluation of produce prescription programs for patients with diabetes and food insecurity for both public and private payers; with a focus on ensuring access for those with greatest need. In practice, a national produce prescription program could be enacted through inclusion of produce prescriptions as a covered benefit, as is already occurring in limited capacities in select Medicare Advantage plans and in several state Medicaid programs, or perhaps through a new program administered by the US Department of Agriculture similar to SNAP or WIC.

### Strengths and Limitations

Strengths of this investigation include use of a validated microsimulation model, incorporation of nationally representative data on individuals with diabetes and food insecurity, and inclusion of parameters based on evidence synthesis of meta‐analyses on effects and costs of produce prescription programs and diet–disease associations, increasing confidence in the results. The model integrated uncertainties in the input parameters through probabilistic sensitivity analyses, and 1‐way sensitivity analyses further supported our primary findings when varying specific inputs and assumptions. Health effects, costs, and cost‐effectiveness were assessed at a lifetime as well as shorter time horizons of 5 and 10 years, from both health care and societal perspectives, providing policy makers a range of plausible outcomes of expanded insurance coverage of produce prescriptions. Potential impacts on specific population subgroups were evaluated and underscore the potential for produce prescriptions to improve health equity if appropriately implemented and targeted.

Potential limitations should be considered. Our simulation cannot prove health and cost effects of a national produce prescription program. Rather, our results provide the most likely health benefits and cost effects, and their underlying uncertainty, based on the best available data and reasoned assumptions. Under extensive sensitivity analyses with varying assumptions, our primary results were robust. Intervention effects were based on the pooled effects of 20 produce prescription programs, which had varying amounts of produce provided, provision methods (food box delivery/voucher/card), geographic locations, patient demographics, and durations; and health effects of produce prescriptions could vary depending on these factors. Because the existing literature does not allow a rigorous assessment of this heterogeneity, our findings should be considered the most plausible average effect based on the best available data, as well as quantifying the uncertainty in this average effect. Further studies can help identify implementation factors and patient characteristics that might influence the efficacy of produce prescriptions. Most studies in our meta‐analysis used a quasiexperimental design without a control group, and additional studies with a randomized design are needed to advance the field. We did not consider the potential health benefits of produce prescriptions for family members of the enrolled patients, possibility underestimating the full health and economic impacts. The published produce prescription studies reported up to 18 months of intervention, and our estimation of sustained programs to 5 years, 10 years, and a lifetime could be either over‐ or underestimates of cost savings, depending on whether the efficacy strengthens or wanes over time. Our model estimated incremental health care costs associated with different health status using a prediction algorithm developed from Medical Expenditure Panel Survey, which excludes people in nursing homes and assisted living facilities who represent a particularly high‐expenditure group. This did not account for potential economic benefits associated with prevention of institutionalization among enrolled patients; and Medical Expenditure Panel Survey also tends to underestimate health care expenditures due to underrepresentation of high‐expense cases and underreporting of health care use.^46^, ^47^, ^48^, ^49^, ^50^ Thus, the health care cost savings estimates from our model should be considered as conservative estimates.

## Conclusions

This study suggests that implementing produce prescriptions nationally for patients with diabetes and food insecurity could improve health, reduce health care costs, and be highly cost effective in the United States. Our findings support the testing, scaling, and evaluation of produce prescription programs for patients with diabetes and food insecurity for both public and private payers with a focus on ensuring access to those with greatest need.

## Sources of Funding

The current work was supported by the National Institutes of Health and the National Heart, Lung, and Blood Institute (2R01HL115189‐06A1; PI, Dariush Mozaffarian). The funding agency did not contribute to the design or conduct of the study; collection, management, analysis, or interpretation of the data; preparation, review, or approval of the article; or the decision to submit the article for publication.

## Disclosures

Dr Mozaffarian reports research funding from the National Institutes of Health, the Gates Foundation, The Rockefeller Foundation, Vail Innovative Global Research, and the Kaiser Permanente Fund at East Bay Community Foundation; personal fees from Acasti Pharma and Barilla; scientific advisory board, Beren Therapeutics, Brightseed, Calibrate, Elysium Health, Filtricine, HumanCo, Instacart, January Inc., Perfect Day, Tiny Organics, and (ended) Day Two, Discern Dx, and Season Health; stock ownership in Calibrate and HumanCo; and chapter royalties from UpToDate. Dr Wong is a member of the US Preventive Services Task Force (USPSTF). This article does not necessarily represent the views and policies of the USPSTF. All other authors declare no conflict of interest in the submitted work. This study used publicly available data from NHANES (https://wwwn.cdc.gov/nchs/nhanes/Default.aspx), Medical Expenditure Panel Survey (https://www.meps.ahrq.gov/mepsweb/), and CDC WONDER (https://wonder.cdc.gov/Deaths‐by‐Underlying‐Cause.html), as outlined in the article. No data are available in addition to the submitted materials. The remaining authors have no disclosures to report.

## Supporting information

Data S1
Tables S1–S5
Figures S1–S8
References^51^, ^52^, ^53^, ^54^, ^55^, ^56^, ^57^, ^58^, ^59^, ^60^, ^61^, ^62^, ^63^, ^64^, ^65^, ^66^, ^67^, ^68^, ^69^, ^70^, ^71^, ^72^, ^73^, ^74^
