# Supplementary material for: Health and Economic Impacts of Implementing Produce Prescription Programs for Diabetes in the United States: A Microsimulation Study
Source: J Am Heart Assoc. 2023 Jul 7;12(15):e029215. doi: 10.1161/JAHA.122.029215 (PMC10492976; doi:10.1161/JAHA.122.029215)
Supplement: Supplementary file 1 — Data S1 Tables S1–S5 Figures S1–S8 References 51 , 52 , 53 , 54 , 55 , 56 , 57 , 58 , 59 , 60 , 61 , 62 , 63 , 64 , 65 , 66 , 67 , 68 , 69 , 70 , 71 , 72 , 73 , 74 [file JAH3-12-e029215-s001.pdf]

Supplemental Material

Data S1. Supplemental Methods

1.1 DOC-M model description

The Diabetes, Obesity, Cardiovascular Disease Microsimulation (DOC-M) model is a probabilistic, dynamic, individual-level, health-state transition model, programmed in R-4.1.0, that jointly projects obesity, diabetes, CVD, and their associated complications for population health and health policy decision-making. The detailed model development process and validation analyses are presented in a separate paper (Kim et al, unpublished data).

The model is populated with individual data from the National Health and Nutrition Examination Survey (NHANES) and accounted for proper survey weights to provide estimates representative of the U.S. population. Each simulated person in the DOC-M model can transit through multiple health states each year: no CVD or diabetes, diabetes without CVD, CVD without diabetes, both CVD and diabetes, and death, plus four CVD-related events (first or recurrent stroke or coronary heart disease [CHD], with an option for revascularization for each) (**Figure S1**). The annual probability of experiencing a CVD event and changing health states for each individual was predicted from validated CVD and diabetes risk prediction algorithms and national mortality data, based on each individual's detailed risk profile at the start of each year, underlying secular population trends, and diverse potential simulated interventions. The model also captures the incidence and prevalence of overweight (body mass index [BMI] ⩾25 and <30 kg/m2) and obesity (BMI ⩾30 kg/m2), based on each individual's dynamic BMI influenced by their lifestyle behaviors and underlying secular age-sex-race/ethnicity-specific national trends. By tracking transitions over time, the model captured changes in body mass index (and thus incident and prevalent obesity) and incidence and prevalent diabetes, CVD, all-cause mortality, quality-adjusted life years, and health care costs at the individual level for a representative population of US adults. The model jointly incorporated parameter uncertainties using probabilistic analyses with 1,000 Monte Carlo simulations drawing from probability distributions for all input parameters. The model combined within-simulation variance (i.e., sampling uncertainty) and between-simulation variance (i.e., parameter uncertainty) to reflect propagated uncertainty in modeled outcomes using an adaptation of Rubin's rule [34]. The source code is freely available at https://github.com/food-price/DOC-M-Model-Development-and-Validation. Subsequent sections describe key input parameters and approaches in more detail (**Table S1**).

The validation analysis demonstrated strong model performance based on both population-risk calibration and individual-risk discrimination for all primary and secondary outcomes for the U.S. population overall and by racial-ethnic groups. Comparing the 15-year model-predicted population risk of primary outcomes among the 2001-02 NHANES cohort with the observed prevalence from age-matched cross-sectional 2003-16 NHANES cohorts, calibration performance was strong based on observed-to-expected ratio and calibration plot analysis. In most cases, Brier scores fell below 0.0004, indicating a low overall prediction error.

1.2 Systematic review and meta-analysis on effect sizes of produce prescription programs.

The intervention effects on dietary habits, BMI, and HbA1c were estimated based on a new meta-analysis of quasi-experimental or randomized controlled produce prescription interventions. We focus on programs that evaluated the impact of continued fruit and vegetable prescriptions over an extended period (more than three months) on fruit and vegetable intake, BMI, and HbA1C. A total of 20 produce prescription intervention programs were identified, including 11 studies in a recent systematic review, 6 more studies from our unpublished analysis of completed produce prescription programs, and 3 more studies identified through Pubmed searches after the screening date of the systematic review (see eTable B1 for detailed characteristics of the included studies). Among the eligible studies, most (17 of 20) enrolled adults with poor cardiometabolic health including diabetes or prediabetes, hypertension, CVD, obesity, or overweight. All programs enrolled participants who were food insecure or at high risk for food insecurity, with a weighted average of 74% being food insecure. In addition, all 10 programs that assessed the effect on HbA1c were implemented among diabetes patients, with 79% being food insecure. The average age of participants across all programs was 54 years, with 69% female, 38.5% non-Hispanic Black adults, and 30.0% Hispanic adults. This population was a bit younger and had a higher percentage of females and non-Hispanic Black individuals than the national population with diabetes and food insecurity from NHANES, where the average age was 58 years, 55.5% were female, and 17% were non-Hispanic Black. However, a recent study from our team evaluating the impact of produce prescription interventions on dietary intake and cardiometabolic risk factors identified no significant evidence of differential effects by age, sex, race/ethnicity, or SNAP enrollment status.

Study-specific effect estimates were pooled using inverse-variance weighted random-effects meta-analysis. The I^2 statistic was used to assess the heterogeneity of included studies, with values <25%, 25 to 50%, and >50% corresponding to low moderate, and high degrees of heterogeneity, respectively. A high level of heterogeneity was observed for fruit and vegetable intake, and HbA1c level (I2>90%), and for BMI, a low level of heterogeneity (I2=22%). Multiple factors could influence the effect size of the produce prescription programs, including inclusion criteria, the dollar amount of food vouchers or food boxes, sample size, duration of the study period, and factors that could hardly quantify such as nutrition education provided, and convenience for accessing the fruits and vegetable purchase point. Due to the small number of studies included for each outcome (13 studies for fruits and vegetable intake, 9 studies for BMI, and 10 studies for HbA1c), and various study quality, we had little confidence to tease out the possible interactive effect of different component of produce prescription and therefore focused on evaluating the average effects across studies.

Based on the weighting factors for each study generated in the meta-analysis, we calculated the weighted mean of the dollar amounts of produce prescriptions provided and average dollar amounts redeemed per capita.

1.3. Estimation of administrative costs of national produce prescription program

We estimated the administrative costs of the national produce prescription program by reviewing the costs of the Special Supplemental Nutrition Program for Women, Infants, and Children (WIC) and the Supplemental Nutrition Assistance Program (SNAP), each of which provides nutrition assistance for eligible individuals. Both programs provide nutrition education in addition to financial benefits, with WIC required to provide specific types and numbers of nutrition education, and nutrition education being optional in SNAP with State flexibility. SNAP provides an average monthly benefit of $130 per person for 40 million participants. The administrative costs of SNAP contribute to 5-8% of total program costs according to pre-pandemic program cost data, including State administrative expenses, eligibility certification, nutrition education, employment and training programs, benefit and retailer redemption and monitoring, payment accuracy, EBT Systems, program evaluation and modernization, program access, and health and nutrition pilot projects. WIC provides an average monthly benefit of $61.35 per mother for 6-8 million participants each year. After excluding a minority of administrative costs related to breastfeeding promotion and education (about 1.8% of total program costs), administrative costs of WIC contribute to about 21.3% of total program costs, including participant eligibility, nutrition education, health care coordination, and referral, drug abuse education, clinic operations, food delivery and warehousing, vendor monitoring, financial management, program integrity, and systems development and operations.

A national produce prescription program may share similar administrative components with both WIC and SNAP on eligibility certification, quality control, employment and training, use of the EBT system or food delivery, benefit and retailer redemption and monitoring, and program evaluation. A national produce prescription program may have greater similarities with WIC than SNAP, as WIC includes partnerships with healthcare organizations and has higher intensity and consistency of nutritional education. Still, the administrative burden and costs could be lower in a national produce prescription program than in WIC, as the former provides only produce rather than multiple categories of foods, does not require income certification, and does not need to establish separate clinical institutions (WIC clinics). We assumed that the administrative costs of a national produce prescription program would be 15% of total program costs, about 2-3-fold higher than the administrative costs of SNAP and one-third lower than the administrative costs of WIC. In sensitivity analysis, we evaluated alternative administrative costs of 8% (upper end of SNAP costs) and 21.3% (WIC costs). We also assumed the costs to be higher in the first year of implementation due to program launching, equal to 50% of food costs (33% of total program costs).

1.4 Estimating healthcare cost savings associated with HbA1c level in the produce prescription program

HbA1c is recognized as an important predictor of healthcare costs for diabetes patients, independent of multiple diabetes comorbidities such as CVD and hypertension.[28,29] Patients with good glycemic control were less likely to treat their diabetes with medication and less likely to

be prescribed insulin,[51] and are associated with a reduced risk of diabetes-related microvascular (neuropathy, retinopathy, and nephropathy) and macrovascular (CHD and stroke) complications,[52-54]

Since the cost prediction algorithm of OCD-M does not intrinsically capture the influence of HbA1c changes in the medical costs of diabetes, we further incorporated additional HbA1c-related cost savings in the model. We assumed that improved HbA1c could reduce health care costs for diabetes patients by reducing costs of diabetes treatment such as diabetes medications, physician visits, and self-testing devices, [51] and costs related to treating microvascular complications (neuropathy, retinopathy, and nephropathy), [52-54] and macrovascular complications (CHD and stroke). To avoid double-counting for the savings of healthcare expenditures related to CVD outcomes, which have already been captured by the fruit and vegetable and BMI pathway, we restricted our estimation to non-CVD-related healthcare costs.

To identify the best available evidence for HbA1c reduction on diabetes-related health care costs, we systematically reviewed studies assessing the impact of HbA1c reduction on diabetes-related health care costs in the US from 2000 through now. Despite diverse data sources, and the scope of healthcare costs, all studies suggested reducing HbA1c is associated with reduced healthcare costs.

We used the evidence from the study by Maureen et al 2020, the most recent available data that provided the cost estimation related to HbA1c reduction. The study suggested that a 1 percentage point reduction in HbA1c was associated with a 13% reduction (or a $736 reduction) in diabetes-related healthcare costs, independent of comorbidity conditions. [55] We assumed that this cost reduction equally applied to both CVD and non-CVD-related diabetes-related costs.

The DOC-M predicts the marginal effect of having diabetes on health care expenditure ($3859.002 per person per year), independent of age, sex, race, CVD, BMI, and blood pressure. The marginal effect of diabetes intrinsically subtracted CVD-related costs from diabetes costs. We estimate that a One percentage point reduction in HbA1c was associated with a 13%*$3859.002 =$501.67 decrease in CVD-independent diabetes-related medical costs per person per year.

Table S1. Key model parameters and data sources for evaluating the health and economic impact of the produce prescription program.

	Value/Mean (SE) *	Primary Source
**Transition probabilities**		
**Developing type 2 diabetes**	Framingham Offspring Study 8-year diabetes risk model	[56]
**Initial ASCVD**	ACC/AHA 10-year ASCVD risk model	[57]
**% CHD vs. Stroke**	Sex-race-specific values (47.4-64.0% vs. 36.0-52.6%)	[58]
**Subsequent CVD**	Framingham Heart Study 2-year risk model for subsequent CVD	[59]
**% CHD vs. Stroke**	Sex-race-specific values (58.0-73.2% vs. 26.8-42.0%)	[58]
**Death from non-CVD/diabetes; diabetes; CVD; CVD or diabetes**	CDC Wonder age-sex cause-specific mortality table	[60]
**% receiving RVSC**	67.3%	[58]
**% CABG vs. PCI among RVSC**	28.9 vs. 71.1%	[58]
**Death from CABG vs. PCI**	1.8% vs. 2.1%	[58]
**Individual HRQOL**	HRQOL prediction model	[31]
**HRQOL decrements with**		
**CHD**	-0.055 (0.011)	[32]
**Stroke**	-0.3 (0.06)	[32]
**Individual healthcare cost**	Healthcare cost prediction model	Our Own Analysis using MEPS Data [25] (**Table S2**)
**Annual cost of**		
**CHD**	10,034 (2,006)	[27]
**Stroke**	15,994 (3,199)	
**CABG**	44,538 (8,908)	
**PCI**	18,477 (3,695)	
**Intervention effects**	Value (95%CI)	New meta-analysis (**Data S1, Table S3**, and **Figure S2, Figure S3 and Figure S4**)
**Fruit & vegetable intake, servings/day**	0.80 (0.45-1.15)	
**BMI, kg/m²**	0.36 (0.16 to 0.55)	
**HbA1c, %**	0.63 (0.28-0.98)	
**Diet-disease etiologic effects**	RR (95% CI)	
**CHD**		
**Fruits**	0.94 (0.91-0.98)	
**Vegetables**	0.95 (0.92-0.98)	
**Ischemic stroke**		[2, 22]
**Fruits**	0.88 (0.83-0.93)	
**Vegetables**	0.83 (0.75-0.93)	
**Hemorrhagic stroke**		
**Fruits**	0.73 (0.62-0.87)	
**Vegetables**	0.83 (0.72-0.96)	

**Policy costs, per person per year**		
**Food costs**	$382 (52.8)	
**Administrative costs,**		**Data S1**, section 1.3
**First-year**	$191 (26.4)	
**Following years**	$57 (7.92)	

Abbreviations: ASCVD, atherosclerotic CVD; ACC/AHA, American College of Cardiology/ American Heart Association; CDC, Centers for Disease Control and Prevention; CVD, cardiovascular disease; CHD, coronary heart disease; RVSC, revascularization, including coronary artery bypass surgery (CABG) and percutaneous coronary intervention (PCI); HRQOL, health-related quality of life; MEPS, Medical Expenditure Panel Survey; SE: standard error.

*: Where uncertainty around input parameters (e.g., cost of CABG/PCI) is not available, we assume 20% of the mean estimate as a standard error to generate parameters for probabilistic distributions.

Table S2. Marginal effects of individual characteristics on estimated annual health care expenditures among U.S. adults aged 40-79 years, based on 73,174 individuals in MEPS 2014-16.

	Mean annual healthcare expenditures (95% CI)	
	**Mean ($)**	**95% CI**
Baseline health care expenditure*	2895	2687, 3102
Age after 40, each year [i.e., age 40 = 0]	+95.1	84.2, 106
Changes in BMI from BMI 28, each kg/m2[e.g., BMI 25 = -3; BMI 30=2]	+41.0	15.1, 66.8
Female vs male sex	+1984	1645, 2323
Race/Ethnicity		
Non-Hispanic White	Reference	
Non-Hispanic Black	-1629	-2113, -1144
Hispanic	-2312	-2705, -1920
Non-Hispanic Other	-1581	-2163, -998
Diabetes	+3842	3167, 4516
High blood pressure	+2101	1634, 2568
Coronary heart diseases	+4711	3834, 5587
Stroke	+4850	3673, 6028

Abbreviations: BMI, body mass index; CI, confidence interval. MEPS, Medical Expenditure Penal Survey.
* Baseline healthcare expenditures capture the average annual total healthcare expenditures for individuals who were aged 40 years, male, non-Hispanic Whites, BMI of 28, and had no diabetes, high blood pressure, coronary heart disease, or stroke. The marginal effects represent additional changes in health care expenditures from the baseline expenditure by the change in one unit of the predictor. For example, with all other predictors unchanged from baseline characteristics (i.e., BMI 28, male, non-Hispanic Whites, and no clinical condition), a one-unit change in age (from age 40 to age 41) would increase annual healthcare expenditures by $95 on average. Thus, the model included the healthcare costs for all conditions not directly captured in the model (e.g., asthma, joint pain, etc.) according to their average costs for a US adult of any given age, sex, race, and cardiometabolic health.

Table S3. Percentages of missing values for individual-level characteristics in the model population.

Key variables	Percentage missing (%)
Age in years	0
Sex	0
Race/ethnicity	0
Educational Level	1%
Family income to poverty ratio	9%
BMI	0
History of cardiovascular disease	3%
Smoking	0%
Total cholesterol	6%
Triglyceride	54%
HDL	6%
SBP	7%
DBP	7%
Family history of diabetes	2%

Abbreviations: BMI, body mass index; DBP: diastolic blood pressure; HDL, high-density lipoprotein; SBP, systolic blood pressure.

Table S4. Characteristics and effect size estimations of the produce prescription programs the meta-analysis.

Studies *	Study Design	Eligibility	Monthly prescription food/dollar amount	Monthly food costs†	Mean duration (months)	Sample Size	Change in HbA1c, %, mean (SE)	Change in BMI, kg/m2, mean (SE)	Change in F&V Intake (servings/day), mean (SE)
**Bihan et al (2012)**[61]	RCT	Low-income adults 18-60 years	$10-40	$10.5	3	302	NR	NR	0.12 (0.29)
**Freedman et al (2013)** [62]	Pre-post	Diabetes patients attending a health center	$15	$9.7	5.5	41	NR	NR	0.54 (0.86)
**Seligman et al (2015)** [63]	Pre-post	Diabetes patients who use food banks	20-25% of monthly food needed per household	$27	6	687	-0.15 (0.06)	NR	0.23 (0.07)
**Bryce et al (2017)** [64]	Pre-post	Diabetes patients	$15	$16	3	74	-0.71 (0.14)	NR	NR
**Cohen et al (2017)** [19]	Repeated measures	SNAP-enrolled adults at a health center	$10	$10	3	177	NR	NR	0.65 (0.14)
**Cavanagh et al (2018)** [65]	Pre-post	Low-income adults with chronic diseases	$28	$33	5	54	NR	-1.1 (0.45)	NR
**Seligman et al (2018)** [66]	RCT	Diabetes patients who use food banks	20-25% of monthly food needed per household	$32	6	568	NR	NR	0.34 (0.17)
**Trapl et al (2018)**[67]	Pre-post	Hypertensive adults with food insecurity	$40	$33.4	3	224	NR	NR	1.60 (0.20)
**Emmert-Aronson et al (2019)** [68]	Pre-post	Patients with chronic diseases, such as CVD, diabetes, or depression, and food insecurity	$40	$29.4	4	49	NR	0.40 (0.25)	1.24 (0.12)
**Feinberg et al (2019)** [69]	Pre-post	Diabetes patients with HbA1c >8%, and food insecurity	Foods for 10 meals/week for household	$24	18	NR	-2.1 (0.42)	NR	NR

**Ferrer, et al (2019)** [70]	RCT	Diabetes patients with HbA1c >9%, and food insecurity	10 lbs of produce	$25	6	29	-1.4 (0.66)	-1.01 (0.6)	NR
**Orsega-Smith et al (2020)**[71]	Pre-post	Food insecure adults attending a pediatrician's clinic	15-26 lbs of produce	$29	12	41	NR	NR	0.63 (0.33)
**Basu et al (2021)**[17]	Pre-post	Low-income adults	$20	$26.6	6	671	NR	NR	0.22 (0.04)
**Veldheer et al (2021)** [18]	Pre-post	Adults with diabetes (HbA1c>7, and with an overweight)	$28-140	$42.1	7	97	-1.3 (0.23)	-0.57 (0.33)	NR
**Hager et al (unpublished data, 2023), 2020 Hartford**	Pre-post	Adult with HbA1c> 6.5%, from low-income community	$60	$48	6	221	-0.15 (0.11)	NR	NR
Hager **et al** (unpublished data, 2023**), 2019 Humana**	Pre-post	Food insecurity with at least one chronic disease	$15	$11.85	8	69	-0.04 (0.244)	-0.22 (0.04)	NR
**Hager et al (unpublished data, 2023), 2014 BCBS**	Pre-post	Adults with a diagnosis of diabetes recruited from health centers in a low-income community	$30 per household member	$58.9	4	76	-0.55 (0.234)	-0.05 (0.15)	2.19 (0.225)
**Hager et al (unpublished data, 2023), 2019 OH**	Pre-post	Adults with diabetes or prediabetes recruited from health centers serving a low-income community	$30 per household member (120 max)	$72.4	10	227	-0.57 (0.128)	-0.45 (0.15)	1.41 (0.22)
**Hager et al (unpublished data, 2023), 2020 OH**	Pre-post	Adults with diabetes or prediabetes from health centers serving a low-income community	$90	$73.3	10	142	-0.36 (0.158)	-0.36 (0.32)	0.70 (0.17)

**Hager et al (unpublished data, 2023), 2020 Weight Watchers**	Pre-post	Adults with overweight or obesity	$50	$31.6	6	425	NR	-0.38 (0.79)	0.49 (0.11)

Abbreviations: CVD, cardiovascular disease; F&V, fruit, and vegetables; NR, not reported; RCT, randomized controlled trial.

[*] All included studies reported changes in fruit and vegetable consumption (in servings per day) pre- and post-intervention, or as a difference in differences between the control and intervention groups. Some studies reported these effect sizes for fruits and vegetables separately, and some studies reported them in aggregate. [†] Values were converted to 2021 US dollars.

Table S5. Estimated age-specific etiologic effects of fruits and vegetables on cardiometabolic outcomes[*]

	Cardiometabolic outcome	Unit of effect	Estimated relative risks (95% CI), by Age					
			**25-34y**	**35-44y**	**45-54y**	**55-64y**	**65-74y**	**75+y**
Fruits	↓ CHD	RR per 100 g/d	0.92<br>(0.87, 0.97)	0.92<br>(0.87, 0.97)	0.93<br>(0.89, 0.97)	0.94<br>(0.91, 0.98)	0.95<br>(0.92, 0.98)	0.97<br>(0.96, 0.99)
	↓ Ischemic stroke		0.83<br>(0.76, 0.90)	0.83<br>(0.77, 0.90)	0.86<br>(0.80, 0.92)	0.88<br>(0.83, 0.93)	0.9<br>(0.86, 0.94)	0.94<br>(0.92, 0.96)
	↓ Hemorrhagic stroke		0.63<br>(0.49, 0.81)	0.64<br>(0.5, 0.82)	0.69<br>(0.56, 0.84)	0.73<br>(0.61, 0.87)	0.77<br>(0.67, 0.89)	0.86<br>(0.8, 0.92)
Vegetables	↓ CHD	RR per 100 g/d	0.93<br>(0.89, 0.97)	0.93<br>(0.9, 0.97)	0.94<br>(0.91, 0.97)	0.95<br>(0.93, 0.98)	0.96<br>(0.94, 0.98)	0.98<br>(0.97, 0.99)
	↓ Ischemic stroke		0.76<br>(0.64, 0.9)	0.77<br>(0.66, 0.9)	0.8<br>(0.7, 0.92)	0.83<br>(0.74, 0.93)	0.86<br>(0.78, 0.94)	0.92<br>(0.87, 0.96)
	↓ Hemorrhagic stroke		0.76<br>(0.61, 0.95)	0.77<br>(0.62, 0.95)	0.8<br>(0.67, 0.96)	0.83<br>(0.72, 0.96)	0.86<br>(0.76, 0.97)	0.92<br>(0.86, 0.97)
			(0.70, 0.88)	(0.71, 0.89)	(0.75, 0.90)	(0.79, 0.92)	(0.82, 0.93)	(0.90, 0.96)

Abbreviations: CHD, coronary heart disease; CI, confidence interval; RR, relative risk.

[*] The detailed methods for reviewing and synthesizing evidence to estimate effect sizes for associations between dietary factors and cardiometabolic endpoints have been reported.[23,24] We utilized evidence from meta-analyses of prospective cohorts or randomized clinical trials evaluating direct associations of dietary factors with coronary heart disease (CHD), stroke, or type 2 diabetes, by age.[2,23,24]

[†] The available evidence suggests an effect of seafood omega-3 on fatal CHD, with less clear evidence for benefits on nonfatal CHD.[72] Because the risk transitions influenced by diet in the CVD-Predict model are for the incidence of a CHD event, with subsequent transitions to death independent of dietary risk factors (see Fig 1), the current analysis will modestly overestimate the benefits of changes in seafood omega-3 consumption.

Supplemental Figures

Figure S1. Conceptual diagram of the model structure for the DOC-M.

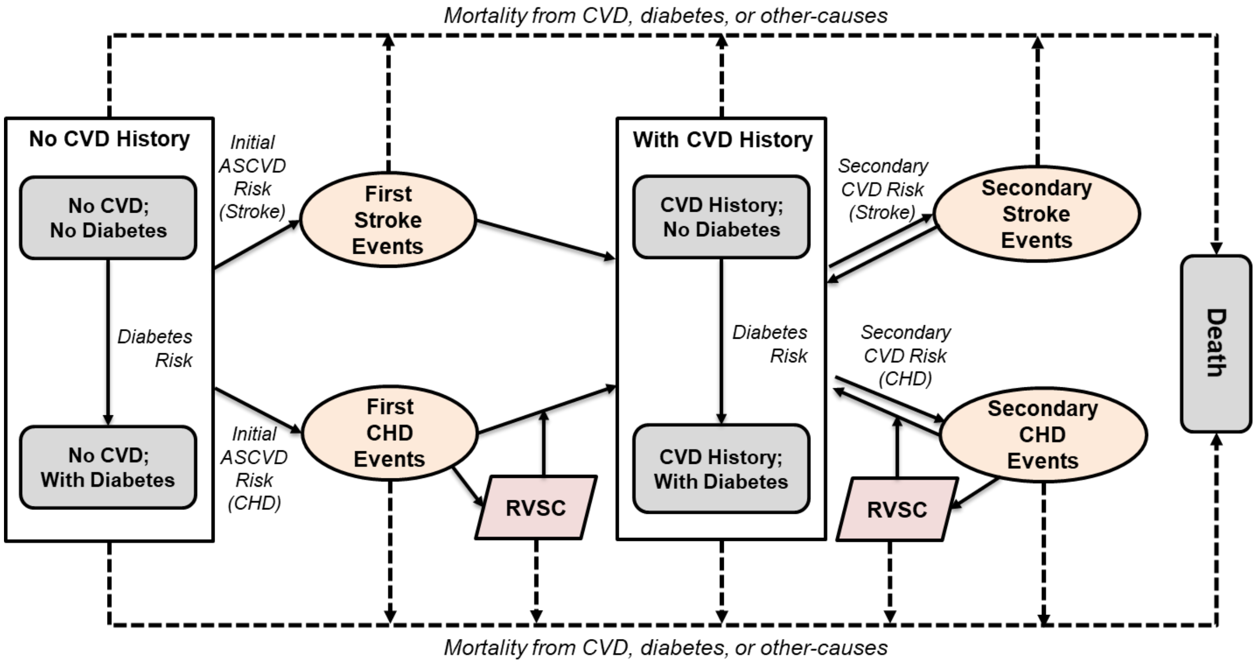

Abbreviation: CVD, cardiovascular diseases; CHD, coronary heart diseases; RVSC, revascularization, including coronary artery bypass surgery and percutaneous coronary intervention.

Note: Figure 1 highlights key transitions from one health state/event to another state/event using solid arrows while dotted-line arrows represent cause- or event-related mortality. Grey rectangles represent five different health states in which individuals can stay throughout modeled periods, while yellow circles show cardiovascular disease events that individuals can experience in any given year. Diabetes was defined as self-reported diabetes or one of four clinical criteria (i.e., fasting plasma glucose level ≥ 126 mg/dl, 2-hour plasma glucose level ≥ 200 mg/dl, Hemoglobin A1c level ≥ 6.5%, or use of diabetic medications) [73]. Individuals with a prior history of CVD events were defined as those who reported experiencing at least one of the four events: angina, stroke, heart attack, and coronary heart disease. For angina, we used the Rose questionnaire criteria [74] or the use of anti-angina medications.

Figure S2. Forest plot for effects of produce prescription programs on fruit and vegetable (F&V) intake.

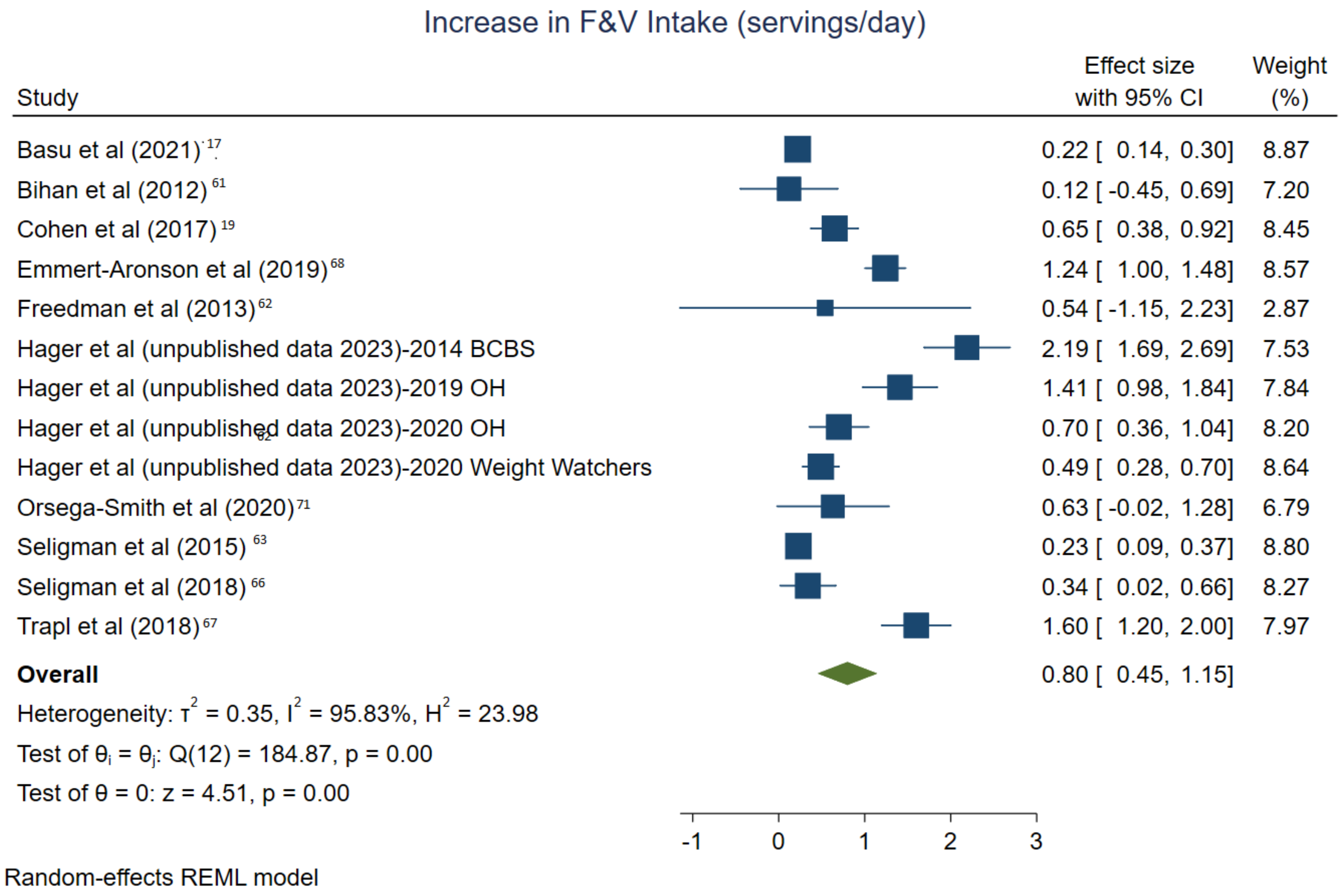

Note: Data were pooled using random-effects meta-analysis. In most studies, combined fruit and vegetable intake was assessed, whereas effect sizes for disease outcomes were available for fruits and vegetables separately, we assumed similar average effect sizes of produce prescriptions on fruit vs. vegetable intake (0.4 servings/day each). The duration of these intervention studies ranged from 3 to 18 months.

Figure S3. Forest plot for effects of produce prescription programs on BMI.

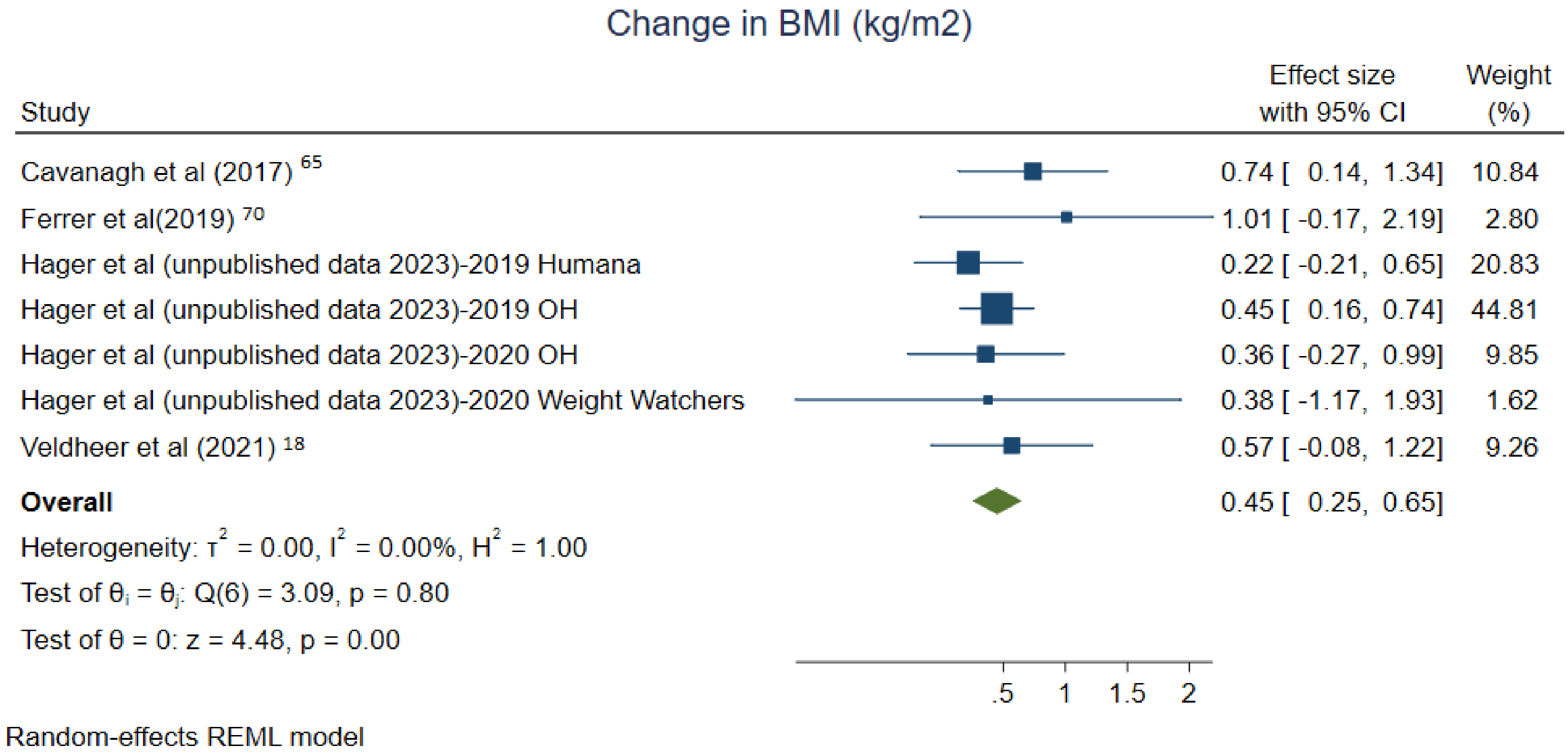

Note: Data were pooled using random-effects meta-analysis. Pooled results are statistically significant even when most of the individual studies were insignificant, due to the improved statistical power from pooling multiple studies

Figure S4. Forest plot for effects of produce prescription programs on HbA1c.

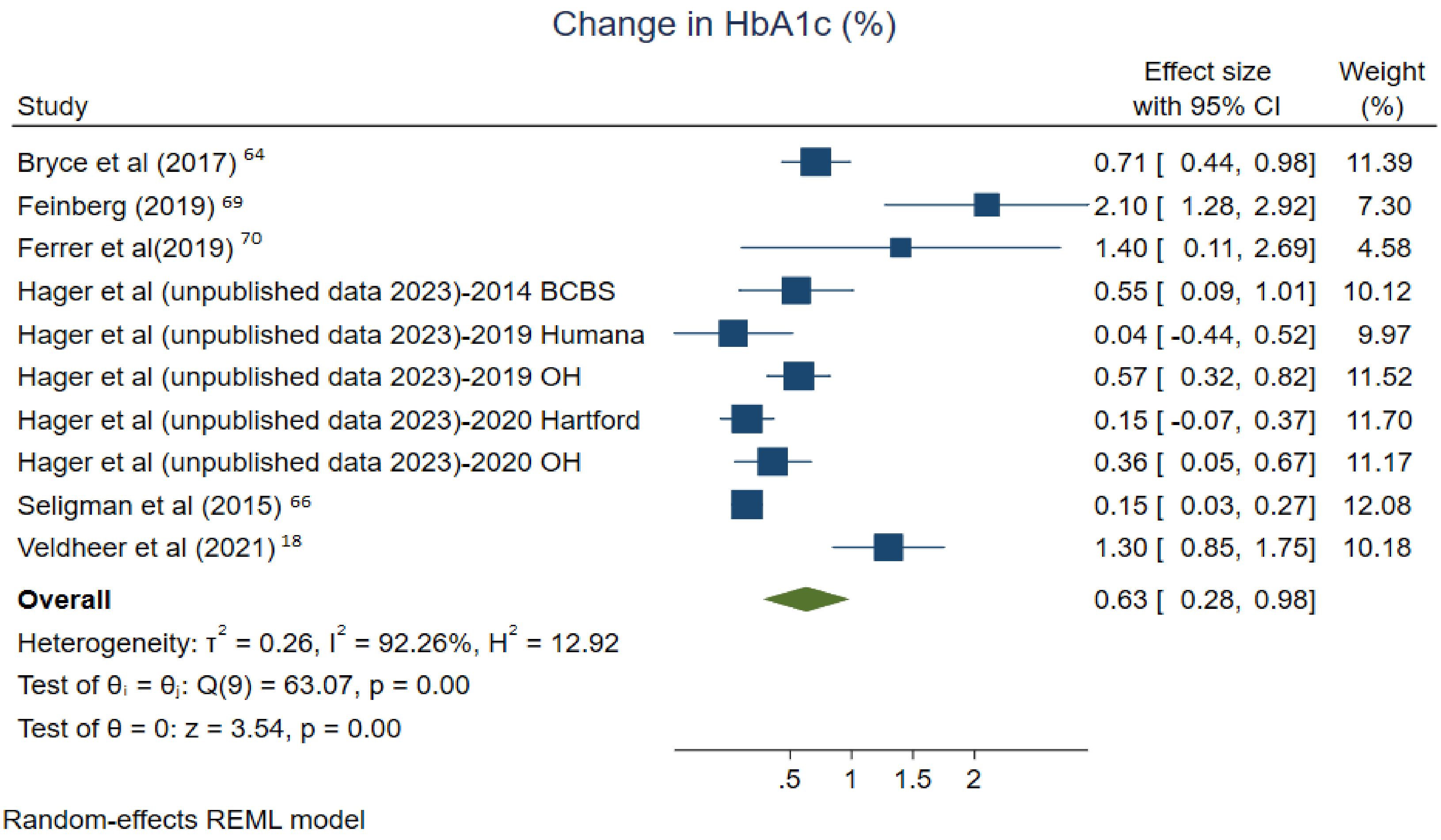

Note: Data were pooled using random-effects meta-analysis

Figure S5. Estimated QALY gains of a national produce prescription program by population subgroups, per 100,000 population, at 5 years, 10 years, and a lifetime of intervention

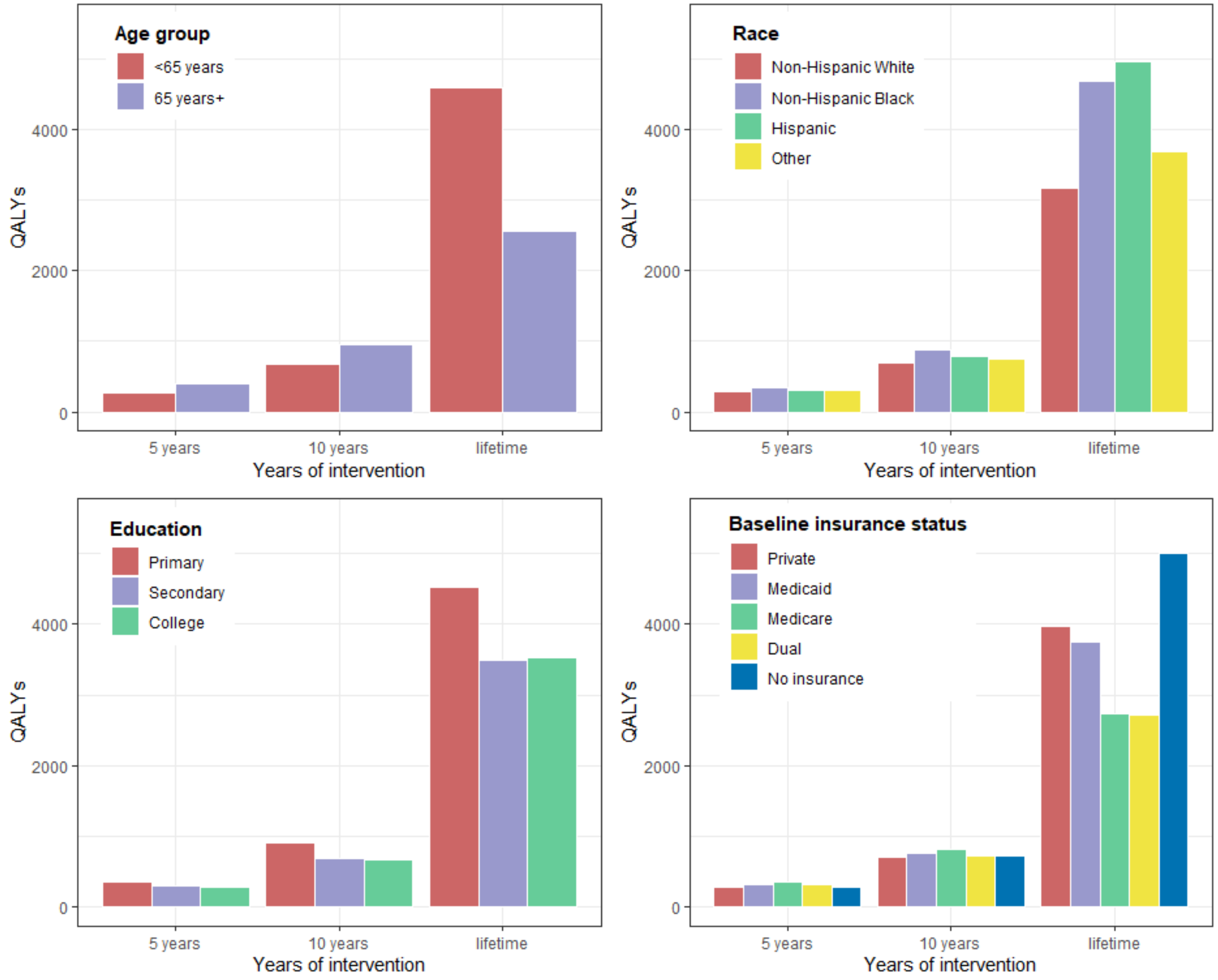

Note: Bars represent the average QALYs averted by produce prescriptions per 100,000 diabetes patients estimated from 1000 Monte-Carlo simulations using the DOCM model, by comparing the identical population (diabetes patients with food insecurity) undergo projections with and without implementing produce prescription. QALYs were discounted at 3% annually. Abbreviations: QALYs: quality-adjusted life years; HIS: Hispanics; NHB: non-Hispanic Blacks; NHW: non-Hispanic Whites; Other includes all other racial/ethnic groups that are not HIS, NHB, NHW.

Figure S6. Estimated healthcare cost savings of a national produce prescription program by population subgroups, per 100,000 population, at 5 years, 10 years, and a lifetime of intervention

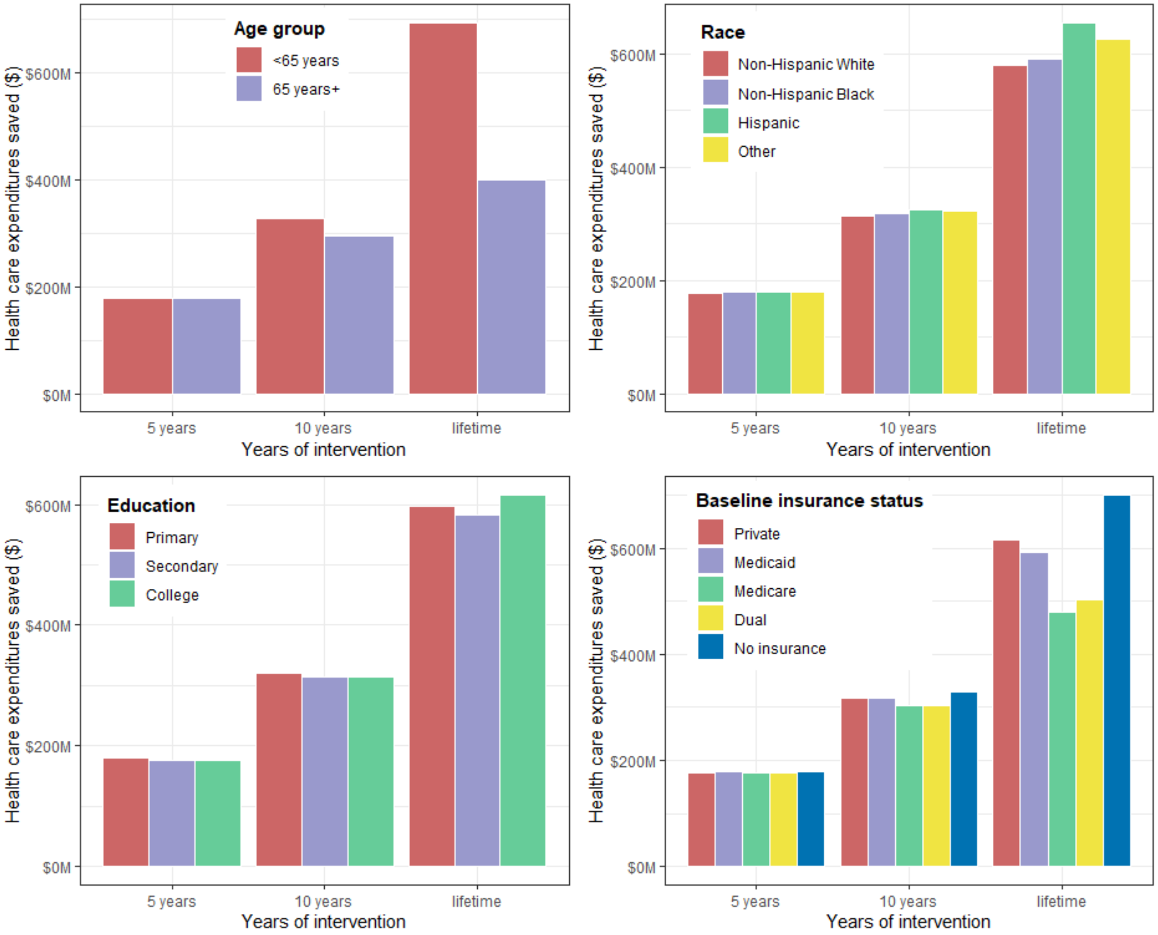

Note: Bars represent the average healthcare costs saved by produce prescriptions per 100,000 diabetes patients estimated from 1000 Monte-Carlo simulations using the DOCM model, by comparing the healthcare costs of identical populations (diabetes patients with food insecurity) undergoing projections with and without implementing produce prescription. The “Other” Race/ethnicity group includes all other racial/ethnic groups that are not HIS, NHB, or NHW.
Abbreviations: QALYs: quality-adjusted life years;

Figure S7. Net changes in costs and net monetary benefits by varying intervention effect sizes on different percentiles of its distribution.

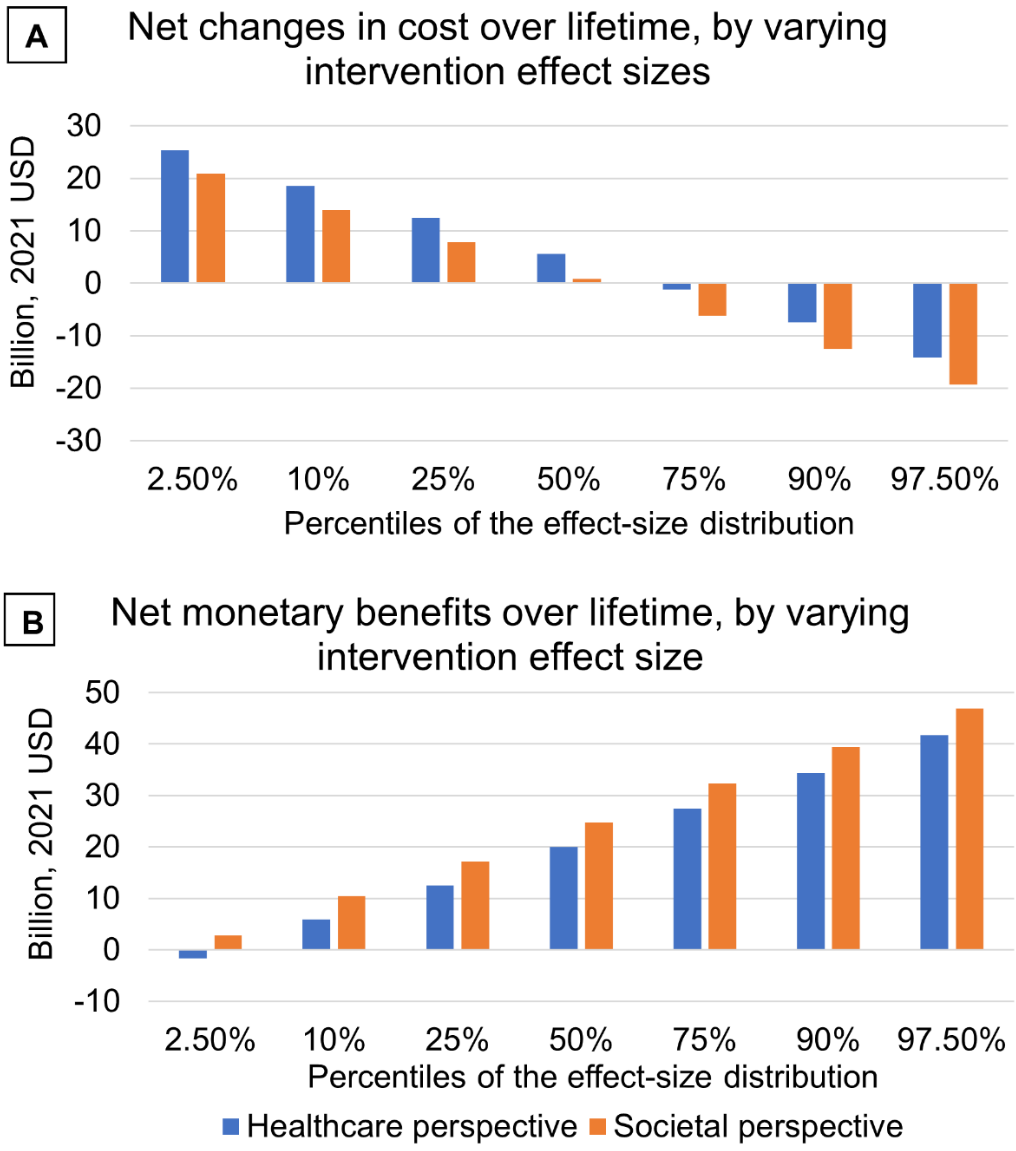

The average intervention effect size and its uncertainties of the produce prescription program on fruit and vegetable intake, BMI, and HbA1c were estimated from a new meta-analysis of 20 produce prescription programs that assessed these outcomes. The 2.5th to 97th percentiles intervention effect sizes are equivalent to an increase of 0.083, 0.17, 0.26, 0.39, 0.53, 0.65, 0.76 servings/day for fruit and vegetables respectively, and reductions of 0.26, 0.31, 0.38, 0.44, 0.51, 0.57, and 0.64 kg/m2 for BMI, and reductions of 0.27%, 0.39%. 0.49%, 0.62%, 0.75%, 0.87%, and 0.97 for HbA1c. Net changes in costs from a healthcare perspective = total intervention costs - healthcare cost-savings by produce prescriptions; Net changes in costs from a societal perspective = total intervention costs - healthcare cost-savings - savings from averted productivity loss. Negative values in the net costs indicate cost savings. The met monetary benefits were calculated as estimated QALYs averted* willingness to pay threshold($100,000/QALY) - net change in costs. A positive net monetary value indicates that the intervention is cost-effective.

Figure S8. Net costs by varying assumptions on administrative costs for program implementation

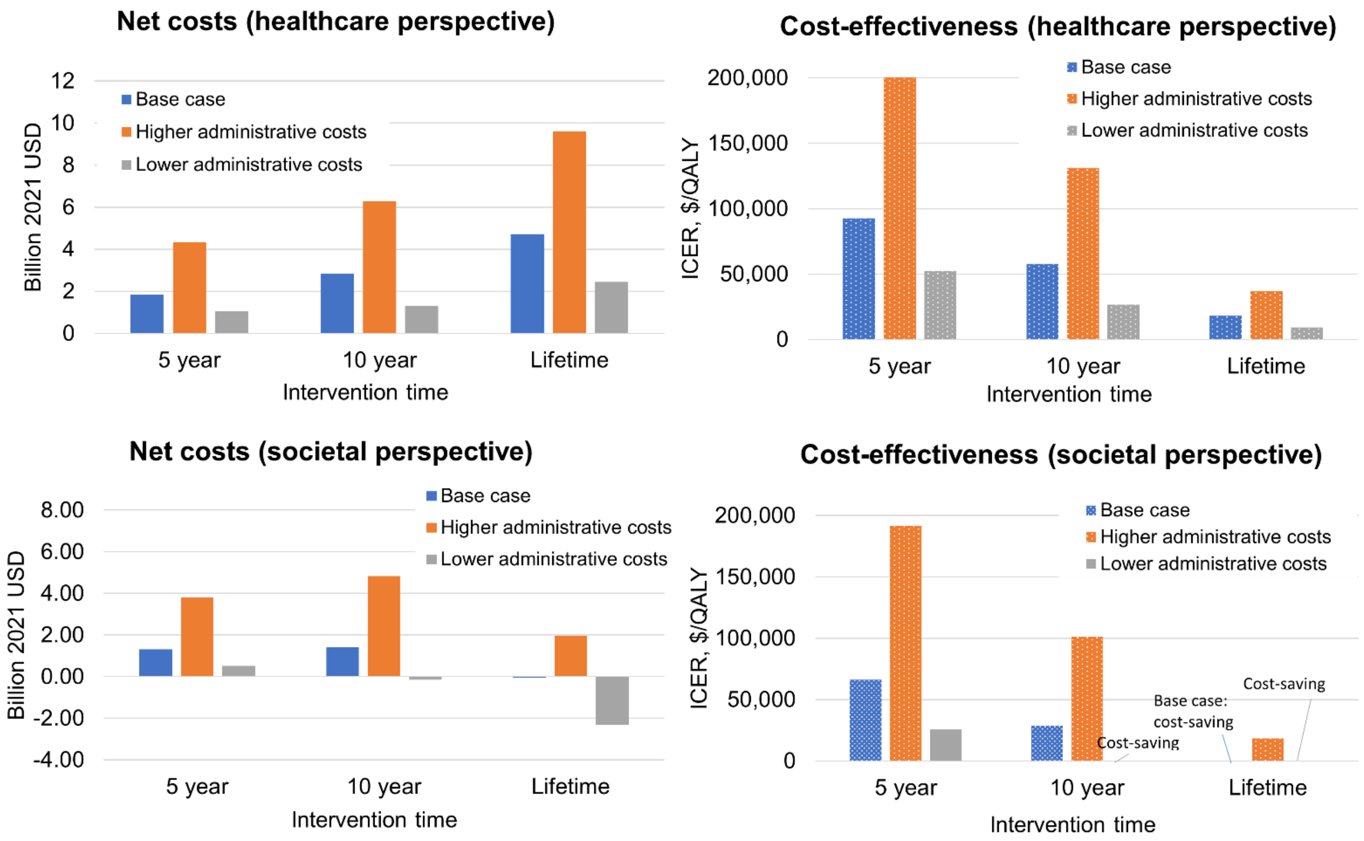

The weighted average monthly incentive costs for the 20 studies for estimating intervention effect sizes were $31.9/month (SE =4.4) in 2021 USD. In the base case, we estimated the administrative costs to be equal to 15% of the annual total program cost or equal to 17.5% of the incentive costs. Also, we assumed the cost is doubled in the program launching stage at the first year of intervention, equal to 30% of total program costs. We additionally varied the administrative costs as a percentage of total program costs, for lower administrative costs at 8% of total program costs and higher at 23% of total program costs, which are also doubled in the first year of intervention.
